# Integrated socio-environmental vulnerability assessment of coastal hazards using data-driven and multi-criteria analysis approaches

**DOI:** 10.1038/s41598-022-15237-z

**Published:** 2022-07-08

**Authors:** Ahad Hasan Tanim, Erfan Goharian, Hamid Moradkhani

**Affiliations:** 1 Civil and Environmental Engineering, University of South Carolina, C206, 300 Main St., Columbia, SC 29082 USA; 2 Center for Complex Hydrosystems Research, Civil, Construction and Environmental Engineering, The University of Alabama, Tuscaloosa, AL USA

**Keywords:** Hydrology, Natural hazards, Hydrology, Civil engineering

## Abstract

Coastal hazard vulnerability assessment has been centered around the multi-variate analysis of geo-physical and hydroclimate data. The representation of coupled socio-environmental factors has often been ignored in vulnerability assessment. This study develops an integrated socio-environmental Coastal Vulnerability Index (CVI), which simultaneously combines information from five vulnerability groups: biophysical, hydroclimate, socio-economic, ecological, and shoreline. Using the Multi-Criteria Decision Making (MCDM) approach, two CVI (CVI-50 and CVI-90) have been developed based on average and extreme conditions of the factors. Each CVI is then compared to a data-driven CVI, which is formed based on Probabilistic Principal Component Analysis (PPCA). Both MCDM and PPCA have been tied into geospatial analysis to assess the natural hazard vulnerability of six coastal counties in South Carolina. Despite traditional MCDM-based vulnerability assessments, where the final index is estimated based on subjective weighting methods or equal weights, this study employs an entropy weighting technique to reduce the individuals’ biases in weight assignment. Considering the multivariate nature of the coastal vulnerability, the validation results show both CVI-90 and PPCA preserve the vulnerability results from biophysical and socio-economic factors reasonably, while the CVI-50 methods underestimate the biophysical vulnerability of coastal hazards. Sensitivity analysis of CVIs shows that Charleston County is more sensitive to socio-economic factors, whereas in Horry County the physical factors contribute to a higher degree of vulnerability. Findings from this study suggest that the PPCA technique facilitates the high-dimensional vulnerability assessment, while the MCDM approach accounts more for decision-makers' opinions.

## Introduction

Coastal regions face various challenges including tropical cyclones, severe storms, shoreline erosion, tsunamis, sea level rise (SLR), and coastal flooding, which collectively lead to more unprecedented adverse consequences on coastal regions’ ecosystems and socio-economic conditions. More frequent occurrences of hurricanes, coastal floods, high tides, and waves deteriorate the coastal ecosystem, and negatively affect economic welfare and people’s health, albeit of different degree and magnitude^1,2^. The ecosystems’ health and the coastal regions’ natural hazards vulnerability have been impacted by complex interactions among the connected social and biophysical subsystems. These social-environmental systems are facing various sorts of challenges and risks such as climate change^3^, population growth and urbanization^4,5^, ecological disturbance^3^, climate uncertainties and discrepancies in urban and rural socio-economic conditions^6^, unsustainable management^7^, and conflicts and fights^8^. Each of these challenges can be the topic of emergent research, which often roots in different types of socio-environmental relationships^9^. Thus, the long-term sustainability of coastal socio-environmental systems are tied to comprehensive and integrated vulnerability assessment of sub-systems, and then collective adaptive planning and management for the future.

The damages caused by recent hurricanes^10^, such as Katrina (2005), Sandy (2012), Matthew (2016), and Irma (2017), highlight the need for the rearrangement of urban planning, natural conservation, and emergency management strategies^11^ and call for more proactive approaches to evaluate the vulnerability of coastal systems through a new lens. This requires a simultaneous and compound analysis of socio-environmental and biophysical aspects^10,12^. Hurricanes are one of the most devastating and cascading coastal hazards, and the system's vulnerability to hurricanes should be investigated in a multivariate approach^13^. Hurricane is composed of destructive multi-events, including heavy rain, storm surge, flood, strong wind, and landslides. Likewise, the complex feedback and simultaneous effects of biophysical and socio-environmental factors on vulnerability of the system requires a multivariate analysis approach, in which the vulnerability index is estimated by the collective effect of all factors. This allows decisionmakers and land planners to look at the vulnerability of the system as a co-existence of all vulnerability factors associated with the various sub-systems in coastal regions^14,15^. These factors, with respect to their quantitative and qualitative characteristics, can be classified into different groups, namely physical characteristics, hydroclimate, environmental factors, and socio-economic perspective and the shoreline vulnerability (Table 1). The shoreline vulnerability, involving coastal forcing factors, also required for a comprehensive coastal vulnerability assessment.

**Table 1:** Summary of literature review for the vulnerability analysis of natural hazard.

Method	Selected criteria	Weighting	Scaling	Amalgamation	Location
System interconnectivity analysis^43^	Total 58 social and biophysical variables were selected based on literature	Participation coefficient	Z-scores	Multiplex network analysis	Canadian arctic region
Fuzzy TOPSIS and Delphi technique^35^	Social, economic and hydrologic	Delphi	Triangular fuzzy number	Fuzzy TOPSIS	South Han River
Spatial trend analysis of Net primary productivity^52^	Climate change, ecological and hydrothermal factors	Equal weight	Normalization	Multiplication of sensitivity and adaptability	Tibetan Plateau
Multivariate spatial clustering technique^53^	Current and future hurricane flood risk, Socioeconomic and ecological factors	Equal weight	Normalization	Risk analysis	East coast, USA
PCA methods^2^	Tornado intensity and societal exposure	F-scale of tornado	Z-scores	Additive method	Texas
Integrated vulnerability analysis^22^	Coastal forcing, characteristics, biophysical and socio-economic	Expert Knowledge based	Equal weight	Additive method	Azores archipelago
Deterministic and probabilistic model^41^	Land cover and elevation			Spatial prediction using sequential Gaussian Simulation	Manhattan, New York
Bayesian belief network^37^	Landuse, hydrological factors and IDF		Expert Knowledge based	Expectation maximization and gradient descent algorithms	Toronto, Canada
ANN and RF^54^	Socio-economic, hydroclimate, Physical			RF to predict the damage cost and vulnerability classification	Southeast U.S
Convolutional Neural network and SVM^42^	Physical and Geological characteristics, Flood historical location as the triggering factors			Spatial prediction using trained CNN and SVM	Shangyou, China
Support vector machine (SVM)^40^	altitude, aspect, slope, curvature, stream power index, topographic wetness index, sediment transport index, topographic roughness index, distance from river, geology, soil, surface runoff, and land use/cover (LULC)	Frequency ratio (FR) method	Normalization of FR	Spatial prediction	Malaysia
Random-forest (RF) and boosted-tree models^39^	Flooded area, Physical characteristics (Elevation, Slope, Distance from the river, Slope length factor, Topographic Wetness Index, Stream power index, Plan curvature), Landuse map, Soil drainage, Geology	Drop of the node impurity for the classification or the substitution estimate for the regression	Weighted sum the predictor importance	Spatial prediction	Seoul, South Korea
Multi‐Criteria Decision Support Systems^25^	Socio-economic, Fatalities, Flood defense system, evacuation system	AHP scale	Weighted sum	Analytic Network Process	Tokai, Japan
Bayesian network^38^	Hydro-geology, Socio-economy, Climate, Flood protection	AHP, constant sum and Entropy	Normalization	Bayesian Network	Chungnam and Chungbak provinces, South Korea
AHP^33^	Physical, Geotechnical and Social	Expert knowledge based	Saaty’s scale	Additive method	Odisha coast, India
Integrated vulnerability assessment^31^	Shoreline forcing, Coastal characteristics, and Socio-economic	Equal weight	Subjective scaling	Adding all sub-index and then normalization	Ireland
Integrated vulnerability assessment^32^	Shoreline forcing, coastal characteristics and socio-economic	Equal weight	Subjective scaling	Gornitz method	Odisha coast
Testing the utility function of factors amalgamation^30^	Exposure, sensitivity and coping	Sensitivity analysis	Min–max standardizations	Six Additive and multiplicative functions	South Korea

Various studies have attempted to quantify the vulnerability of coastal systems during the last few decades by combining various sets of parameters and doing single- and multi-variate analysis (Table 1). Almost all these studies follow some basic steps including scaling, weighting, and combining elements from one or more categories of coastal vulnerability factors. However, each study has used a unique method at each step. These methods are discussed in the “Coastal vulnerability assessment method and index” and their advantages and shortcomings are discussed.

### Coastal vulnerability factors

Most studies described in Table 1 have evaluated the coastal systems’ vulnerability by focusing on factors from biophysical factors (coastal forcing and physical characteristics groups), such as hurricane pressure, wind velocity, precipitation intensity, coastal flood risk, slope, elevation, land use/land cover, proximity to valuable structures, mean tide ranges, significant wave height, surge height, morphological erosion etc. (e.g. Refs.^8,9^). Coastal vulnerability evolves beyond just the magnitude of hazards. As suggested, vulnerability assessment should target identifying exposed communities that are not only susceptible to the coastal hazards but also more sensitive to the damages from these hazards^16^. To render a comprehensive vulnerability framework, a multi-dimensional framework, which combines a set of physical, socio-economic, and ecological factors, is required to achieve this goal and enhance conventional approaches^16–20^. The socio-environmental factors are less employed for vulnerability assessment of coastal natural hazards^9,14,20–23^. Socio-economic factors must be considered to represent the adaptive capacity of exposed coastal systems to natural hazards. Recently, many studies (e.g., Refs.^18,24,25^). have tended to incorporate human and ecosystem dimensions in their vulnerability assessment framework with respect to the impacts of coastal environmental variability and hazards on populated coastal regions The socio-economic factors involve social status (income, wealth, and education), cultural aspects, risk perception, political conditions, and institutions for assessing the efficiency of disaster responses, sustainable livelihood options, and post-disaster recovery plans^26^. The demographic characteristics (race, gender, ethnicity) and health care systems that can provide more information about community resilience facing natural disasters^19^. Aggregation of exposure with socio-economic^23^ and ecological factors can lead to an integrated hazard assessment framework and fill the gaps. Therefore, this study aims to aggregate the exposure of socio-economic, ecological with relevant hazard stimuli from hydroclimate, physical vulnerability classes to develop a multi-variate coastal vulnerability assessment framework and provide a novel and informative integrated index. In addition to these factors’ considerations of shoreline vulnerability must be assessed in order to support coastal defense systems because human actions and oceanic forces continuously exert pressure on the shoreline^27^.

### Spatial heterogeneity and uncertainty of weights in MCDM

Spatially homogenous weights in vulnerability functions of MCDM lead to underestimation of importance and underrepresentation factors, such as adaptive capacity, which varies over large spatially distributed area, and over time as well. Spatial heterogeneity of weight stems from geographical and socio-economic diversity in different regions; thus, should be reflected in the spatial factors’ weight^28^. To get the final CVI, and for amalgamation of the factors into one index, the indices from each vulnerability group and their relative weights in MCDM need to be combined. Traditionally, two approaches in geospatial analysis are used, including (1) additive methods (summation of the consisting factors^29,30^, and (2) multiplicative factor amalgamation formula^29^. While using this formula, there still exist a gap between a theoretically obtained vulnerability index i.e., CVI and the actual degree of vulnerability in a system. In the MCDM approach, the values of factors within and among vulnerability groups should be combined, which historically has been done by assigning equal weights^31,32^ or performing surveys to find relative importance of factors and their corresponding weights (e.g., AHP^33^). Using equal weights for the additive factor amalgamation method to obtain the final CVI is not able to prioritize most sensitive vulnerability factors and groups^34^. The gap is the consequence of the fact that existing approaches assign spatially homogenous weights within and over a particular region. It is important to verify the subsequent vulnerability index and the spatial consistency of the vulnerability function for constant and varying weights throughout the region and how this alters the overall results. Still, there is a need to capture the effect of varying and uncertain weights on vulnerability maps and perform a stochastic estimation of validation of maps to reduce the uncertainty associated with a coastal vulnerability index estimation. For this purpose, this study aims to develop a heterogeneous spatial weighting method for multi-criteria decision-making and preform uncertainty analysis of corresponding weights to form a prudent vulnerability index for various spatial scales and extents.

### Coastal vulnerability assessment method and index

First, for the sake of simplicity and consistency, let’s call the vulnerability product and introduced indicator by previous studies as Coastal Vulnerability Index (CVI). Current vulnerability assessment methods are mainly developed by some sorts of a Multi-Criteria Decision Making (MCDM) approach. MCDM method can be engaged to obtain the relative importance among the chosen variables based on either subjective or objective weighting technique. MCDM such as Analytical Hierarchy Process (e.g. Refs.^25,33^), Fuzzy Technique for Order Performance to Ideal Solution (TOPSIS) (e.g. Ref.^35^), and Delphi technique^35^ can be engaged. Alternatively using statistical models like Principal Component Analysis (PCA) (e.g., Refs.^2,36^), Bayesian belief network (e.g., Refs.^37,38^, or by leveraging machine learning models (e.g. Refs.^39,40^), deterministic inundation modeling (e.g. Ref.^41^,), convolutional neural network (e.g., Ref.^42^), and network analysis (e.g., Ref.^43^) can be used for obtaining CVI (Table 1).

The MCDM methods have been used widely for qualitative and quantitative evaluation of decision criteria under different management alternatives (Table 1). After selecting the decision criteria, the MCDM methods follow three major steps of weighting, scaling, and amalgamation. In subjective weighting often selecting, prioritizing, and evaluating criteria are associated with biases arising from humans’ perception and inadequate support of information and evidence. Similarly, in absence of definite preferences and expert or decision makers judgments, objective weight determination methods are also reliable, but these are based on mathematical models. The MCDMs’ objective weighting is a data driven approach that relies on large amount of information to prioritize the vulnerability hotspots, but potentially disregards the importance of rest of information^44^. This issue can be addressed by using multivariate statistical approaches and Machine Learning (ML) algorithms, such as PCA.

ML models are powerful tools to visualize and sort out communicable information from rich datasets. PCA method aims to reduce the dimensionality of datasets and at the same time minimizes information loss. This method has been used for assessing socio-economic and environmental vulnerability^45–47^, flash flood vulnerability^18,28,31^, and urban environmental vulnerability^48^. Besides that, fine scale CVI analysis has several challenges including presence of missing values requires advanced spatial interpolation method, the normalization of factors requires a continuous scale rather than a discrete scale to capture precise degree of vulnerability^49^. One shortcoming of applying PCA to large spatial datasets is how one should deal with missing values. To overcome this issue, the Probabilistic Principal Component Analysis (PPCA) applies an Expectation–Maximization (EM) algorithm to estimate missing values^50^. Therefore, PPCA can be preferred in spatial vulnerability studies, since the PPCA can represent continuity of spatial datasets along with importance of information. Still, very few studies attempted to assess the coastal vulnerability using PPCA method, especially at smaller grid levels.

Current CVI development approaches e.g., Refs.^2,15,21,32,33^ mainly rely on analyzing biophysical hazard drivers, with the final CVI consisting of geographical and hydroclimate factors to determine the degree of coastal vulnerability. However, coastal vulnerability is also impacted by socio-economic and environmental conditions. Thus, a more comprehensive CVI should simultaneously combine information about socio-economic and ecological aspects of a coastal area along with vulnerability from biophysical hazard drivers^9,16,18,19,22,23,27,31,36,49,51^. Moreover, the factors that contribute to the vulnerability of coastal land and shorelines are different in nature and should be selected accordingly. After selecting a group of vulnerability factors, their information and values should be combined into a single index. Traditional methods mainly rely on subjective weighting methods which are associated with uncertainty in weight assignment which is biased by experts’ judgement and experience. Finally, most of the previous vulnerability assessments have not been truly validated against data from past events, nor cross-validated by other methods.

Considering the limitations of existing methods, two methods: MCDM and PPCA, are utilized in this study to establish an integrated CVI. To perform coastal vulnerability study in a more comprehensive way, a wide list of socio-economic, environmental, geographical and hydroclimate factors are gathered. These factors are then hand-picked for MCDM, or selected for PPCA through statistical importance. The uncertainty associated with selecting appropriate weights for factors is handled using objective weighting and sensitivity analysis for MCDM and data driven objective weighting for PPCA. Because weights may change over space and time, the spatial heterogeneity of weights among different vulnerability groups is explored by sensitivity analysis. In addition, CVI assessment using the PPCA method has a unique potential to deal with missing geospatial values, which have historically been an obstacle for determining the spatially varying importance of factors and creating fine scale vulnerability maps. Finally, the integrated CVIs, estimated by MCDM or PPCA, are validated against observed coastal hazard vulnerability and historical damages to justify their competence in predicting vulnerability.

In summary, the first objective of this study is to develop an integrated framework for the vulnerability assessment of coastal regions by including factors from four general groups of (Hydroclimate, Physical, Socio-Economic, and Ecological), and a unique fifth group for shoreline vulnerability to natural hazards. The second objective is to perform uncertainty analysis of the spatial MCDM and heterogeneous spatial weighting in MCDM to evaluate the rationality of the obtained CVI over various spatial scales and extents. The third objective is to apply a data driven geospatial PPCA method for coastal vulnerability assessment. For this purpose, 20 vulnerability indicators in five vulnerability groups are picked to determine the spatial vulnerability of the coastal counties of SC. Finally, past coastal hazard inundation maps and costs of fatalities are compiled to validate the CVIs obtained from both methods.

## Methodology

The coastal vulnerability concept demonstrates which degree of vulnerability of a socio-environmental systems exposed to coastal hazard drivers, and it depends on a complex feedback between biophysical hazard drivers and the socio-environmental exposures^55,56^. Earlier concepts of vulnerable places to natural hazard in geospatial analysis focused on geographical hotspots where the likelihood of natural hazard events are high^20^. However, more recent definition is extended to include the impact assessment of environmental components in order to reflect the adaptive capacity of a community. This study acquired datasets from various sources (Table 2) to determine the combined effects of factors from physical, hydroclimate, ecological and socio-economic vulnerability groups.

**Table 2:** Sources of data acquired for coastal vulnerability analysis.

Sl	Vulnerability group	Indicators	Product	Web source
1	Hydroclimate	No. of coastal hazard events	NOAA Storm event database	NOAA (https://www.ncdc.noaa.gov/stormevents/)
2		Hurricane track density	National Hurricane Center (NHC) National Oceanographic Atmospheric Administration (NOAA)	NHC (https://www.nhc.noaa.gov/data/hurdat)
3		Surge height	NOAA SLOSH model Maximum Envelopes of Water (MEOW). For historical surge peak and their locations, MOMs (Maximum of MEOW) composite images	NOAA (https://slosh.nws.noaa.gov/)
4		Rainfall intensity	Global Precipitation measurement (GPM)	GPM (https://gpm.nasa.gov/)
5		Sea level rise	NOAA SLR viewer	https://coast.noaa.gov/slr/
6	Physical	Landuse	USDA land cover map	https://datagateway.nrcs.usda.gov/
7		Available water storage	USDA 10 m resolution soil map	https://datagateway.nrcs.usda.gov/
8		Elevation	US Department of Agriculture (USDA) 30 m resolution Digital Elevation model	https://datagateway.nrcs.usda.gov/
9		Distance from coast		
10	Socio-economic	Social Vulnerability Index (SoVI)^45^		
11		No. of Historical and Archeological structures (NHAS)		
12		Cost of fatalities	NOAA storm event database	https://www.ncdc.noaa.gov/stormevents/
13	Ecological	Species richness	SC Gap Analysis Project^57^	^57^
14		Shellfish harvesting	SCDHEC	https://apps.dhec.sc.gov
15		Turtle	SCDHEC	
16	Shoreline	Rate of shoreline change	US Geological Survey (USGS) National Assessment of Shoreline Change Project dataset^58^. The Linear Rate of Regression (LRR) method dataset engaged in this study	https://pubs.usgs.gov/of/2005/1326/gis-data.html
17		Tide range	NOAA tide gauges	https://tidesandcurrents.noaa.gov/map/index.html
18		Significant wave height	National Data Buoy Center	https://www.ndbc.noaa.gov/
			MetOcean	https://app.metoceanview.com/hindcast/
19		Coastal slope	NOAA coastal bathymetry	https://maps.ngdc.noaa.gov/viewers/bathymetry/
20		Beachfront stability	SCDHEC	

This study presents the development of a multi-variate vulnerability index with emphasis on two main aspects of costal vulnerability, biophysical and the socio-environmental vulnerability. The biophysical vulnerability factors refer to those which describe geophysical and hydroclimate characteristics of a coastal system over an extended time period. The socio-environmental vulnerability aspect consists of social, economic, and ecological features. Here, the socio-environmental vulnerability factors represent the demographics and ecological characteristics of a coastal system, such as SC’s coastal regions. Thus, the proposed CVI for coastal systems can (1) measure the degree of vulnerability where the system is exposed to biophysical hazards, and (2) measure the degree of socio-economic and ecological vulnerability of exposed places. Furthermore, shorelines in coastal systems have their own distinct vulnerability characteristics since the vulnerability of the shoreline is mainly associated with oceanographic forces. A total of 15 factors are selected to represent the coastal vulnerability and 5 factors are chosen for shoreline vulnerability (Table 2).

**Figure 1:**
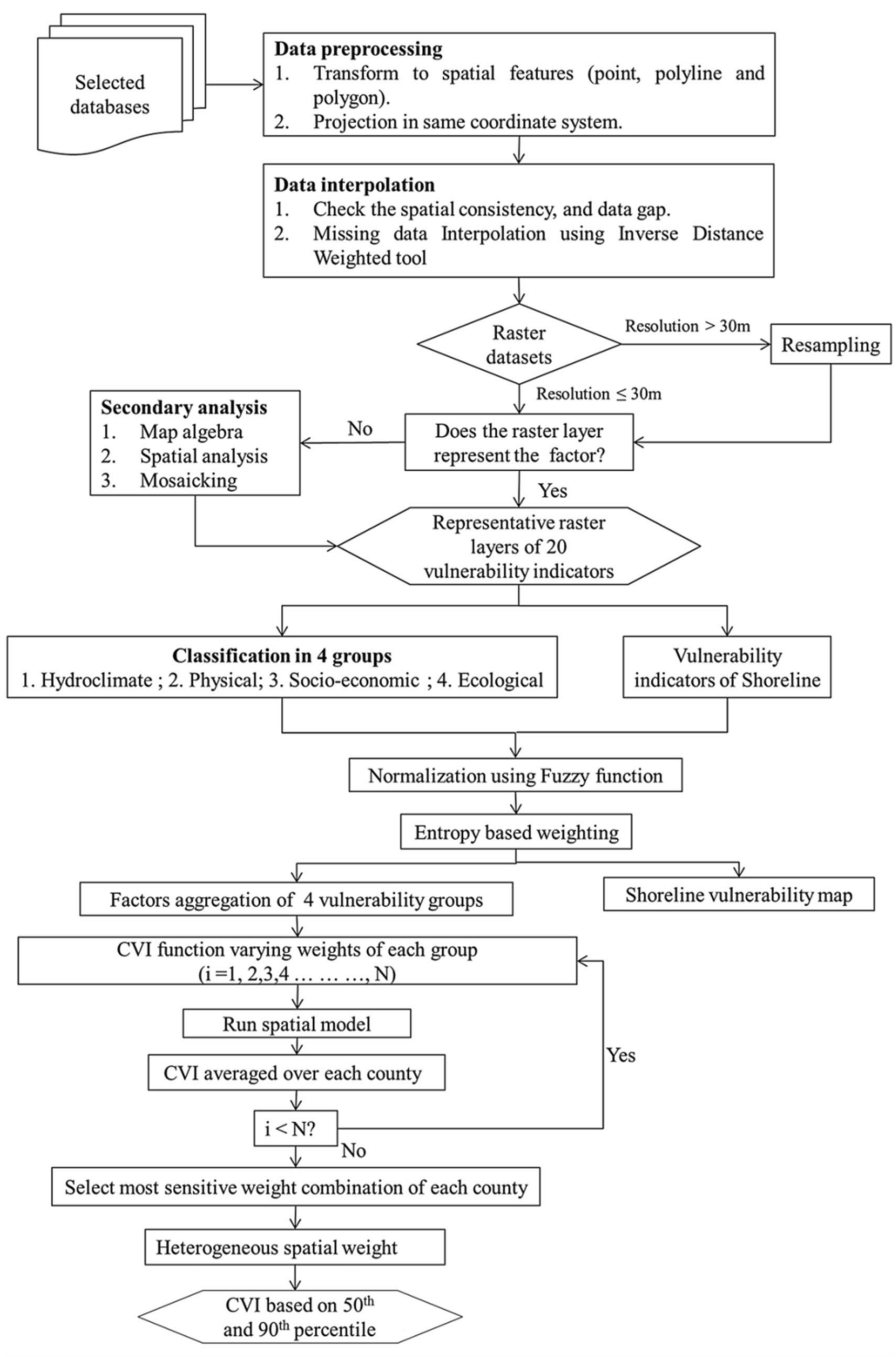
Schematic of the MCDM framework for integrated coastal vulnerability analysis.

**Figure 2:**
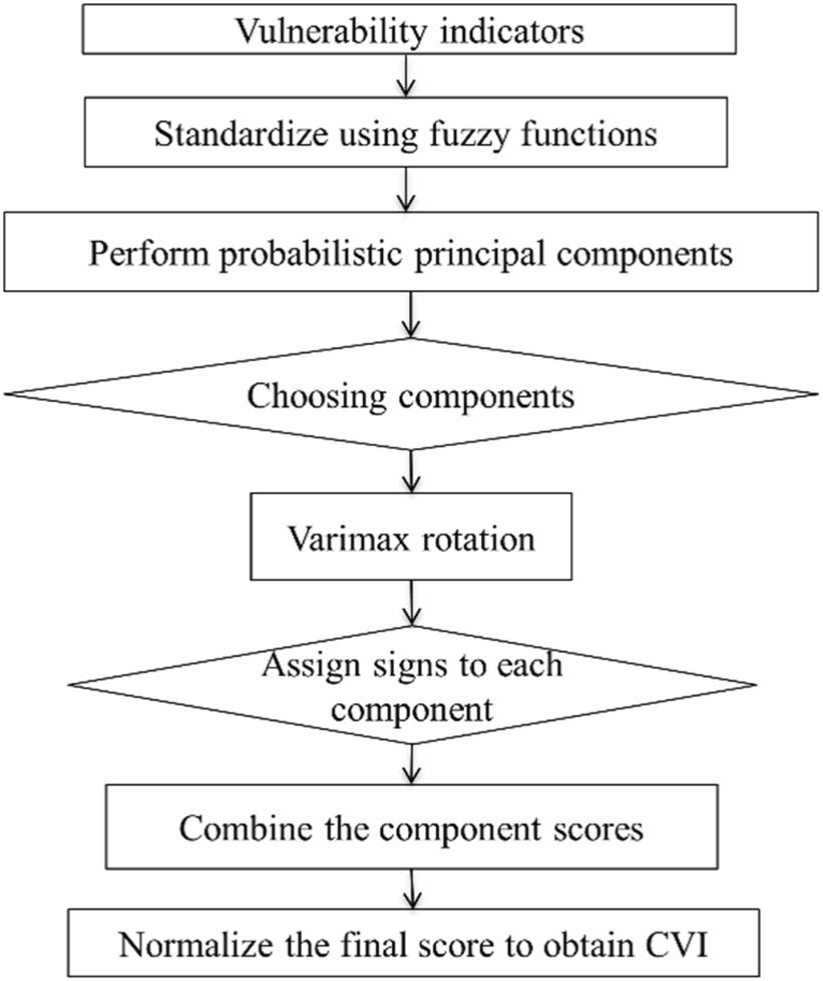
Probabilistic Principal Component Analysis based vulnerability analysis flowchart.

This study propose formation of a new CVI concept based on two different approaches (Figs. 1, 2), MCDM (“Multi-criteria decision-making”) and PPCA (“Probabilistic principal component analysis”). For this, each raster layer consists of about 69 million cells with 30-m resolution over the study region. Fuzzy logic-based normalization and entropy weighting techniques have been used in MCDM to estimate the relative importance of the spatial factors for different vulnerability groups. The overlay and sensitivity analysis are conducted using R packages and ArcGISv10.5. The primary steps needed to obtain a CVI using MCDM methods are presented as follows:

1. Relevant vulnerability factors are selected. After collecting data of relevant spatial factors, these factors are assigned to their representative groups.
2. All data is converted into Geographic Information System (GIS) format. Then, all GIS datasets are converted into raster layers, such that small scale spatial changes can be visualized and studied, using a consistent projection system (here we have used WGS 1984 Web Mercator Auxiliary Sphere).
3. The spatial data interpolation is done if the data is required to be transformed from point shapefile to raster datasets. ArcGIS Inverse Distance Weighting (IDW) Interpolation tool has been used to interpolate the missing values for MCDM. The PPCA method can deal with the missing information using its internal probability model. All geospatial datasets are maintained/generated at a similar spatial scale, here 30 m, to use consistent normalization and factor amalgamation process.
4. The degree of vulnerability of each factor for the MCDM method is determined by Fuzzy logic-based normalization approach (“Scaling with Fuzzy membership function”). This method converts the values of all vulnerability factors to a similar and comparable scale of 0–1.
5. The importance of various spatial factors in a vulnerability group is quantified by the entropy-weight. Multiplying the weights of each factor with the fuzzy normalized layer and summing up all the factors in a vulnerability group results in the vulnerability index of a group.
6. After getting each group’s vulnerability index value, an additive vulnerability function is applied to combine the vulnerability index of all groups into a unique final CVI.
7. The weights associated with vulnerability groups’ index is analyzed with sensitivity analysis to find the ranking percentile of weights for each vulnerability group. For example, CVI-50 and CVI-90 represent the 50th and 90th percentile weights, respectively. In other words, CVI-50 represents a normal vulnerability situation, whereas the CVI-90 depicts a severely venerable scenario.
8. Finally, in order to validate estimated CVIs, a validation dataset has been obtained from Sentinel-1 satellite post processed flood hazard data inventory as well as historical events’ cost of fatalities. The validation datasets are compared with the CVI-50, CVI-90, and later PPCA methods, to evaluate the accuracy of vulnerability assessment using each method.

### Scaling with Fuzzy membership function

After preparing the raster datasets and determinants’ information, Fuzzy set theory is applied to scale the spatial factors based on their importance. While, the Boolean logic accepts binary values for the classification probability, fuzzy set theory considers the probability of a factor as a membership scale between 0 to 1^59^. This study exploits fuzzy logic and its associated functions for factor normalization. Various studies have categorized vulnerability factors into dissimilar ranges to define the degree of vulnerability in a discrete scale. Collective consequences of underestimating or overestimating the degree of vulnerability and its factors in a discrete scale may lead to underestimation or overestimation of the actual vulnerability value and class. Fuzzy logic-based method significantly improves the skill of normalization over min–max, Z-scores, and other traditional normalization methods^60^. For sub-class scale vulnerability analysis, fuzzy-based normalization quantifies the importance of a factor as a continuous function, whereas conventional normalization techniques require reclassification of the data histogram that reduce the natural variability. The main advantages of using fuzzy logic-based factor normalization are the ability to determine the degree of vulnerability using a non-linear data transformation function, the reduction in the uncertainty of discrete scale factor normalization, and the precise indexing of a vulnerability group after summing up all the factors using additive factor amalgamation^49,60–62^.

The relative importance of a pixel value, individual factor vulnerability, will be assigned by a fuzzy membership index. The fuzzy membership function assigns 0 to a non-member pixel, and 1 to a full member. For the values between 0 to 1, three fuzzy functions: (1) Fuzzy large, (2) Fuzzy small, and (3) Fuzzy linear are used to transform the factor value to an index value (Table 3). Equations (1) and (2) show the Fuzzy large (${\mu }_{1}\left(i\right)$) and Fuzzy small ${(\mu }_{2}\left(i\right))$ functions. The Fuzzy linear membership function uses a linear transformation between the lowest value of a histogram, as the minimum limit of zero, and the highest value of a histogram, as the maximum limit of one. The Fuzzy linear function is applied where each quartile range in histogram has equal or uniform distance.

1 $${\mu }_{1}\left(i\right)=\frac{1}{1+{\left(\frac{i}{f2}\right)}^{-f1}},$$

2 $${\mu }_{2}\left(i\right)=\frac{1}{1+{\left(\frac{i}{f2}\right)}^{f1}},$$

where *i* is the raster value of a certain vulnerability indicator, *f1* and *f2* are respectively spread and midpoint from the raster histogram. Fuzzy large function is applied when the range between each quartile is unequal and Fuzzy small function is chosen where vulnerability indicator has a negative correlation with overall vulnerability. Whenever the histogram and frequency distribution of a vulnerability factor are found bimodal or random, the break values categorize the data distribution into five vulnerability classes: high, moderate high, moderate, moderate low and low vulnerable (Table 3). For the fuzzification of the elevation factor, the flooding area of SLR and storm surge is considered as a function of the elevation.

**Table 3:** Rationale of chosen vulnerability indicators and assigned Fuzzy functions.

	Factor	Fuzzy function	Range	Vulnerability class	Rationale
Shoreline	Rate of shoreline change (m/year)	Fuzzy linear and Fuzzy small		High	Shoreline erosion cause land loss
				Moderate high	
				Medium	
				Moderate low	
				Low	
	SWH (m)	Fuzzy large	1.4–4.6		Extreme wave height result faster coastal inundation
	Tide range (m)	Fuzzy large	2.5–3.7		When storm surge arrives in high tide, the tide-surge interaction produces higher storm surge height than a shoreline section having lower tide height. Thus, high tide range increases the surge height probability
	Beachfront stability	Fuzzy small	Stable	Low	Unstable beachfront are more vulnerable to coastal hazard
			Unstable	Very low	
	Coastal slope	Fuzzy small	0–0.06	High	Mild coastal slope enhance coastal flood risk
			0.06–0.14	Moderate high	
			0.15–0.26	Medium	
			0.27–0.56	Moderate low	
			0.57<	Low	
Hydroclimate	No. of coastal hazard events	Fuzzy large	0–166		The numbers of coastal hazard events shows hazard previous footprint on different counties
	Cyclone track density (Wind speed in 30 km radius)	Fuzzy linear	0–51.45		High cyclone activity on a coastal section indicates high vulnerability
	Surge height (m)	Fuzzy linear	0–8		When storm surge height increases the area of coastal inundation will be more
	Rainfall intensity (mm/h)	Fuzzy linear	0.28–0.42		High rainfall intensity can be considered as more vulnerable
Ecological factors	Shellfish harvesting area (km^2^)	Fuzzy large	0.2–1109		Larger shellfish harvesting areas indicate more sensitive ecosystems. Coastal hazards have negative consequences on areas of shellfish harvesting. The higher the area of shellfish harvesting, the higher the chance of damages caused by a coastal hazard to fisheries community
	Turtle sites (km)	Fuzzy large	1–36		Presence of large number of turtle sites is high ecological vulnerability indicators
	Species distribution	Fuzzy large	0–241		Higher the species richness the ecosystem is more vulnerable to coastal hazard
Socio-Economic	SoVI	Fuzzy linear	1–5		High SoVI indicates an area is more vulnerable and less coping capacity
	NHAS	Fuzzy linear	0–430		Historical and Archeological site faces great risk of vulnerability because the life span of structural durability already expired in many places
	Cost of fatalities ($Million)	Fuzzy linear	0–4.32		The higher the cost of fatalities the higher the degree of vulnerability
Physical	Elevation (m)	Fuzzy small	0–0.3	High	Coastal vulnerability decreases with increasing elevation
			0.3–0.6	Moderate high	
			0.61–2.99	Moderate	
			3–7.99	Moderate low	
			8<	Low	
	Curve number	Fuzzy linear	16–45	Low	Landuse having high curve number shows higher potential of generating rainfall runoff and more vulnerable
			46–62	Moderate low	
			63–74	Moderate	
			74–82	Moderate high	
			82–95	High	
	Average water content in soil (cm)	Fuzzy small	0–54.04		High water content in soil increase flood vulnerability by reducing the infiltration capacity
	Distance (km)	K means clustering and Fuzzy Small	0–21.44	High	Proximity to coast increases the level of vulnerability to places and infrastructures
			21.45–41.9	Moderate high	
			42–63.9	Moderate	
			64–87.9	Moderate low	
			88–118.5	Low	

Table 3 lists the assumptions used to categorize each vulnerability component into 5 vulnerability classes. Jenks-optimization-based natural break method is applied to obtain the five categories of the vulnerability: high, moderate high, moderate, moderate low, and low. Shoreline changes are assessed in terms of erosions and depositions, using USGS data. The shoreline change rate can be caused by erosion or accretion. While accretion is not considered a vulnerable situation, its rate is excluded from the estimation of shoreline changes by setting 0 as the margin of Fuzzy linear function. The shoreline erosion rate within 0–5 m/year in histogram is considered for the fuzzification. Afterward, the Fuzzy small function is applied to the shoreline erosion rate in order to relate higher rate of erosion with higher vulnerability values. Then, the rate of shoreline change is classified into five vulnerability classes, and a Fuzzy small function estimates corresponding vulnerability index. The coastal slope histogram has a random distribution and Jenks optimization based natural break method is applied to classify the coastal slope (Table 3).

The elevation map is categorized into different vulnerability classes based on susceptibility of flood caused by storm surge height and SLR with respect to the morphology of the area. In general, the low-lying lands adjacent to coast are more susceptible to flooding. To represent the impact of SLR, the digital elevation model (DEM) maps are categorized based on NOAA-SLR projections to identify impacted areas by different SLR projections. The projected SLR in SC coast is anticipated to be around 0.1–0.6 m by the year 2100^63^. Hence, inundated area projected by NOAA under the scenario of 0.1–0.6 m SLR are identified as vulnerable areas to SLR for this study (Table 3). About 28.6% of total coastal area, including waterbodies, will be likely to be flooded by 2100 under the 0.6 m SLR (Table 3). The Map Algebra tool of ArcGIS is engaged to identify the vulnerability class of each pixel of SLR susceptible area. The area inundated by 0.3 m of SLR is regarded as a highly vulnerable area based on its elevation under more probable coastal floods. The area inundated by 0.3–0.6 m SLR is considered a moderate-high flood risk zone as they will be less affected by SLR and by considering their relative elevation. The area in DEM corresponding to the surge height of 0.61–3 m is defined as moderately vulnerable (Table 3). The historical highest recorded height of a surge in SC coast is about 8 m; thus, the area with elevation above 8 m are assigned as low vulnerable areas, as they are less susceptible to coastal flooding caused by storm surges (Table 3).

The region’s soil type and land cover maps are obtained from gSSURGO soil maps to classify the hydrologic soil groups (Table 2). The impact of landuse on coastal vulnerability is represented by SCS curve numbers determined based on USDA guidelines (USDA 1999) and classified using Jenks optimization based natural breaks methods into vulnerability classes. A Euclidean distance map is generated to classify the area into five vulnerability zones based on distance.

### Multi-criteria decision-making

#### Entropy-based weighting method

There are a variety of objective weighting algorithms available including TOPSIS^64^, entropy concept, Dominance^65^, and Maximin^66^. Here, Lexicographic ordering technique or Entropy-based weighting^67^ has been used in this study to assign weights due to its capability in assignments based on quantitative inhomogeneity among the multiple vulnerability indicators. The entropy-based weighting is assigned to the vulnerability indicators to get the vulnerability of five groups (Table 2). After using fuzzy membership function to show the degree of factors’ value membership with the regards to its vulnerability, the performance matrix of *j* number of factors is normalized using Eq. (1).

3 $${P}_{ij}=\frac{{r}_{ij}}{\sum_{i=1}^{m}{r}_{ij}},$$

where, *i* (1 to *m*) is the index or raster value after fuzzification of a vulnerability factor, and *j* (1 to *n*) is the number of factors in each sub-group. *P*_*ij*_ implies to the normalized payoff of factor *i* under *j* criterion of vulnerability. For each vulnerability sub-group, the entropy (${E}_{j}$) of normalized performance is determined using Eq. (2):

4 $${E}_{j}=-k\sum_{i=1}^{m}{P}_{ij}\cdot \mathrm{ln}{P}_{ij}; where: k= \frac{1}{\mathrm{ln}m}.$$

After calculating the ${E}_{j}$, the entropic weight (${W}_{j}$) is estimated by normalizing the values over the *j* criterion (Eq. 3).

$${W}_{j}=\frac{{d}_{j}}{\sum_{j=1}^{n}{d}_{j}};$$

$$where: {d}_{j}=1- {E}_{j}.$$

5 $$\sum_{j=1}^{n}{W}_{j}=1.$$

#### Vulnerability aggregation

In this stage, four vulnerability groups obtained using objective weighting are joined using intragroup weighting. The vulnerability index of each group, e.g., Physical Vulnerability Index (PVI), Ecological Vulnerability Index (EVI), Socio-Economic Vulnerability Index (SEVI) is calculated using an additive weighted vulnerability function. Vulnerability index of each vulnerability group is determined using Eq. (6)

6 $$SI= \sum_{j=1}^{n}{{P}_{ij}\times W}_{j},$$

where *SI* refers to vulnerability index of each group (*HVI, PVI, EVI, and SEVI*) and $${W}_{j}$$ is the weight obtained using Eq. (5). Here, SI results an index within a range between 0 to 1 because both performance index ($${P}_{ij})$$ and entropy weight ($${W}_{j})$$ in Eq. (6) have corresponding values within a range between 0 to 1. Equation (6) has The CVI is then can be estimated based on a set of intragroup weights (*a*_*k*_*, b*_*k*_*, c*_*k*_*, and d*_*k*_) which are for *k* county (Eq. 7).

7 $$CVI=HVI\times {a}_{k}+PVI\times {b}_{k}+EVI\times {c}_{k}+SEVI\times {d}_{k}.$$

HVI is the Index resulting from hydroclimate vulnerability, PVI is the Index resulting from combining all physical vulnerability indicators, EVI is the Index resulting from ecological vulnerability indicators, *SEVI* is the Index resulting from socio-economic vulnerability indicators. The intragroup weights of are determined using a sensitivity test for different combinations of weights in a range between 0 to 1. The sum of the intragroup vulnerability weights is maintained 1. In this study, HVI, PVI and EVI have 30 m spatial resolution whereas the SEVI is derived at census block level. The CVI-50 and CVI-90 vulnerability map has resolution 30 m. The PPCA map is rendered at 1 km spatial grid resolution.

#### Sensitivity of weights

The sensitivity analysis is performed by enumerating the weights of each group of vulnerability, i.e., *EVI, HVI, PVI* and *SEVI* for different counties. The weights are changed between 0 to 1 with 0.05 intervals, while the sum of weights is maintained at 1, for each county (Eq. 7). Total 730 different combinations of weights were tested to identify the different percentiles of vulnerabilities and their associated sets of weights.

Two vulnerability maps are aimed to develop based on average and extreme conditions of vulnerability. The average and extreme conditions are represented with 50th percentile weights of each county (CVI-50), and CVI-90 for 90th percentile weights of each county respectively. Thus, based on Eq. (7), the weights of CVI factors are spatially varying from one county to another to account the heterogeneity of spatial weights and factor importance. The shoreline section has no other overlapping of vulnerability group, and its CVI is similarly calculated using Eq. (6). The shoreline section is analyzed separately, and then integrated with the coastal land section using “Mosaic” tool in ArcGIS. Mosaic tool is used to combine two or more neighboring raster datasets into a single entity. However, before joining both land and shoreline part both sections are renormalized in 0–1 scale using min–max standardization to have a consistent CVI definition on both sections.

### Probabilistic principal component analysis

PCA is a multivariate statistical method to transform a set of uncorrelated factors to correlated factors, as one principal component (PC), by performing an orthogonal transformation. First, PC maintains the highest possible variance, while the next PCs have the following variances from the constraint. The PCA makes linear combination of the actual factors at reduced dimension that allows a better interpretation of given factors in terms of their importance. The PCA is widely used for dimensionality reduction to obtain a simplified and consolidated index (e.g., Refs.^36,44,50,68^). PCA perform multivariate analysis based on eigenvector. In spatial analysis, the reduced number of factors, components of PCA, are the factor values for each pixel. After transformation of the original factors, PCA assigns a score and loadings for each factor to form new PCs. The loading is the weight of transformation for converting the original factor values into a component score. The probabilistic principal component analysis (PPCA) is a multivariate data analysis technique that combines classical linearized projection and probability model. The extended PCA model^50^ and applied maximum likelihood procedure based on Gaussian probability model allow the PCA method to estimate the missing data in observation datasets.

Prior to PCA analysis the Kaiser–Meyer–Olkin (KMO) test is performed to measure the sampling adequacy. *KMO* criterion ranges between 0–1, in which a sample with *KMO* > *0.5* provides satisfactory result. The datasets used in this study provides a KMO of about 0.72, which shows the reasonable performance of data for PPCA analysis. Another pre-processing criterion is the Bartlett's test of Sphericity. In this test the level of significance with *p*-value should be less than 0.5 for PPCA analysis. The datasets result of 0 in Bartlett's test of Sphericity indicates the sample can be used for PPCA analysis. The PPCA method has also been used to develop a data driven CVI in this study. This method has been originally adopted from Ref.^36^ and has been modified and enhanced here as it is shown in Fig. 2 and described as follows:

1. First, the Spearman correlation coefficient is estimated for all the vulnerability indicators using Fuzzy normalized raster datasets. The correlation coefficient is required to see the highly correlated input variables. Then, the PPCA is performed using the fuzzy normalized input variables.
2. Using PPCA, five principal components are retained using Kaiser criterion^69^. Based on the criterion, the components with eigenvalues greater than 1 are retained.
3. To decrease the number of variables which are used for the vulnerability assessment, the varimax rotation method has been applied. The varimax rotation maximizes the sum of the variances between the squared loadings. Correlations between variables and factors are referred as 'loadings.' So, smaller sets of variables have higher factor loadings, while the remaining variables will get a lower factor loading. This causes varimax rotation to retain only a few key variables in estimation of the CVI.
4. In this step, the influence of the resulting components on coastal vulnerability are interpreted by assigning signs to each component. The positive or negative signs have been added manually to each of the components based on their dominant (the effect of high-loading factors in each component) influence (negatively or positively) on the overall vulnerability. When a higher level of a high-loading variable demonstrates low vulnerability or vice versa, the corresponding factor’s score is adjusted by multiplying it by − 1, for example further distances from coast decreases the coastal vulnerability. When low or high values of a factor influence the coastal vulnerability in the same way (e.g., higher track density increase the coastal vulnerability), the absolute value of the corresponding factor is used.
5. The final CVI is then determined by combining the selected component scores and the explained variance in a weighted sum function^36^.
6. Finally, obtained CVIs from the PPCA method are normalized using min–max standardizations to represent vulnerability values in a range of 0–1.

### Sampling the validation datasets

In order to validate the final CVIs from MCDM and PPCA methods, and to assess their agreements with actual degree of vulnerability, information about historical flooding events is obtained from Sentinel-1 imagery, a geo-big data platform, is used to retrieve a total of 23 Level 1 Ground Range Detected Sentinel-1 Synthetic Aperture Radar (SAR) images. The imageries are acquired during hurricane season of Atlantic Ocean, May–November of the year 2016–2019. Sentinel-1 VH/VV polarization band is used in this study to detect the flooded pixel. In order to prepare the satellite information for the validation of coastal vulnerability, several preprocessing steps are done, (1) updating orbit metadata, (2) thermal noise removal, (3) radiometric calibration (backscatter intensity), and (4) terrain correction or orthorectification using SRTM 30 m DEM. Further, the unitless backscatter intensity images were transformed and reproduced into calibrated, for standard SAR image representation by normalized backscattering coefficient ($\sigma$) values in decibel unit. Further, the ESA's SNAP Sentinel-1 toolbox was used for incidence angle correction and speckle noise reduction by using an adaptive sigma Lee filter (7 × 7). Following^70^ in SAR images a threshold between flooded and non-flooded pixel is calculated. A reference image (R) for representative of dry period is selected for each year, then the absolute difference between reference image (R) and flooded image (F) is determined following Eq. (8). The threshold to identify the flooded pixels is estimated using Eq. (9) ^70^

8 $$d=\left|float (F)\right|-\left|float (R)\right|$$

9 $${\mathrm{F}}_{\mathrm{P}}< \mu \left|d\right|- {k}_{f}*\sigma \left|d\right|.$$

Here, $$\mu$$ and $$\sigma$$ represents mean and standard deviation from absolute difference image ($$d)$$. $${k}_{f}$$ is a coefficient and 1.5 is selected based on recommendations^70^.

Finally, a composite band by clustering SAR images of each year between 2016 and 2019 is formed with an algebraic expression in GEE with a condition that shows the cumulative flood probability of each year. The surge height information was also overlayed to determine the flood severity. Thus, each pixel of the composite images is considered as the flood susceptibility condition on that geographical coordinate. A total of 340 random pixels were sampled to validate the final vulnerability map of SC coast. Additionally, the socio-economic validation of CVI was done by using the cost of fatalities from NWS storm event database. Total 53 locations were used to cross-validate the socio-economic vulnerability.

## Case study

### Study area

**Figure 3:**
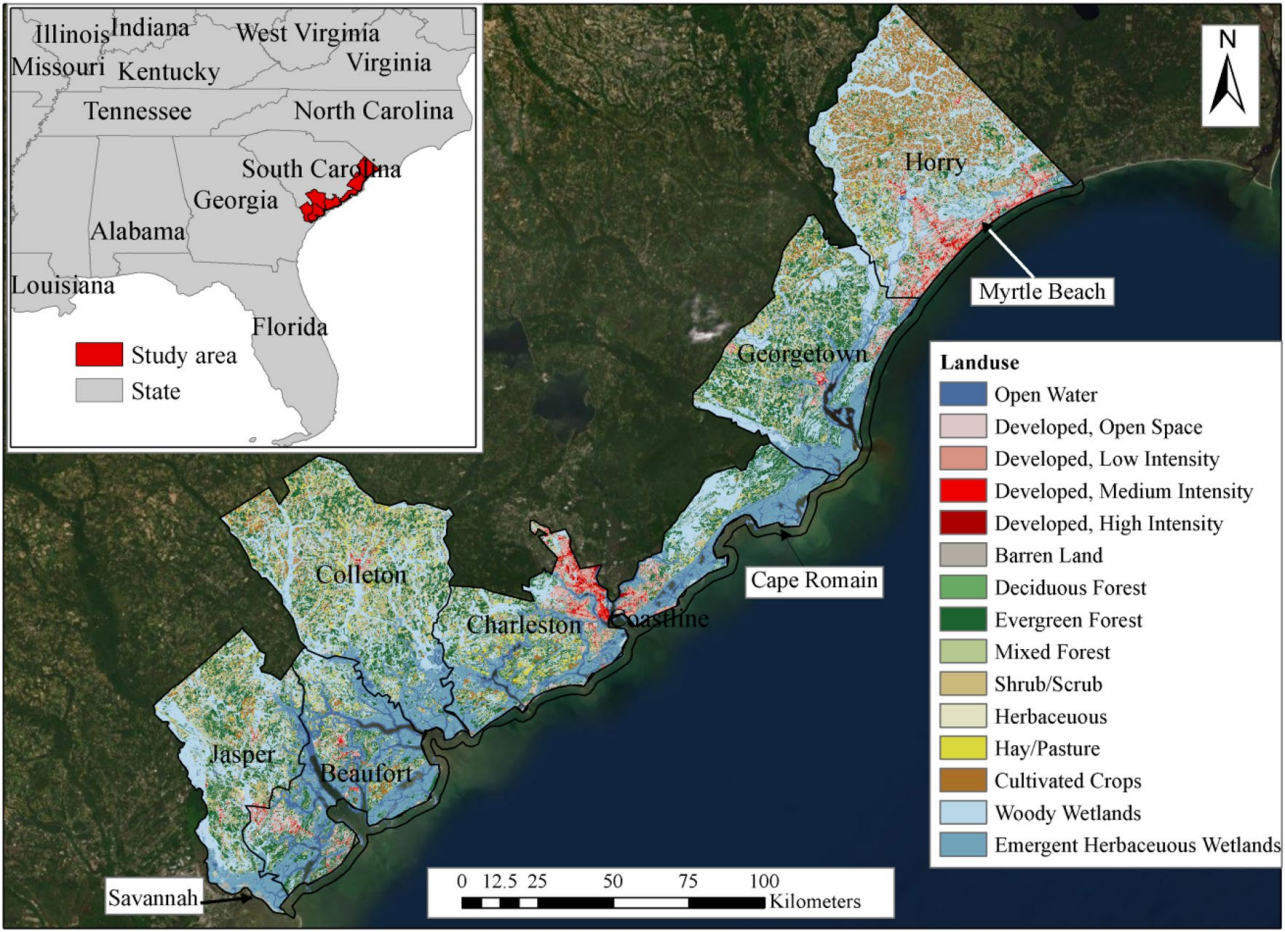
Location of study area in USA (inset map) and the land use types of the South Carolina coast.

The performance of proposed methods for assessing the compound vulnerability of coastal system to natural hazards, the coastal counties of South Carolina (SC) have been selected (Fig. 3). All the figures which contain maps, including Fig. 3 and later vulnerability maps, have been generated originally for this research using ArcGIS v10.5 software (https://www.esri.com/en-us/arcgis/products/arcgis-desktop/overview). The six counties (226 census blocks), Jasper, Beaufort, Colleton, Charleston, Georgetown, and Horry together have a total area of about 15,113 km^2^ and the coastline length of about 475 km with North Atlantic Ocean. A buffer zone of 1 km along the shoreline is considered for the shoreline vulnerability analysis. The SC coastal plain is indeed a vulnerable region considering the frequent hurricanes, SLR, and compound tidal and inland floods. Back in 1980s, the average tidal flooding was about 4 events in a year for the City of Charleston. Due to SLR and numerous storms in recent years, the city is facing more frequent and more severe flooding events. During 2016 and 2017, the tidal flood days was recorded 50 and 46, respectively, in Charleston^71^. The tide along the coast ranges between 1.5 and 2.1 m, with semidiurnal tidal variation and tide cycle duration of about 11.5–12.5 h^72^. Observed SLR data from NOAA shows an increasing trend over the past century, about 3.8 ± 0.2 mm/year at Springmaid Pier and 30 ± 3 mm/year at Charleston^73^. Rich cultural heritage, long history of Civil War, and beautiful beaches transformed the coastal region of the State, in particular the City of Charleston and Myrtle Beach, to major destinations for tourists from all around the world. This historic core earns $8 billion annual revenue from visitors and contains 4th busiest container port in the East coast, USA. The City of Charleston alone brings about 4–7 million tourists annually to this region^74^. However, after hurricanes and floods in past few years, the coastal region of SC is experiencing 10–17% reduction in total number of visitors (USACE 2020). According to US Census Bureau (2021), SC coastal counties have population densities ranging from 18 (Jasper) to 175 (Charleston) people per square km, average poverty rates ranging from 11.9% (Charleston) to 20.1% (Colleton), percentages of population above 65 years ranging from 17% (Charleston) to 28.6% (Georgetown), percentages of disabled people under 65 ranging from 6.7% (Charleston) to 12.6% (Horry), median household income ranging from $36 k (Colleton) to $72 k (Beaufort), and percentages of uninsured population ranging from 13.1% (Charleston) to 19.4% (Jasper). These numbers collectively indicate that concerns over SC’s coastal societies are inherently spatial in nature and its socio-demographic characteristics change greatly over the coastal counties. This fact puts disadvantaged coastal communities more in danger of receiving damage and natural hazards risk, calling for a more integrated coastal hazard assessment framework and future adaption policy. The SC coast is a sanctuary of six national wildlife refuges (NWR) covering an area of about 580 km^2^ and shelters 291 of different species^75^. The hydrologic process in NWR mainly depends on tides and marshes^76^. By 2100, SLR of approximately 0.53 m can submerge 51.4% Cape Romain NWR in SC, and in turn 52 ecological species will be in high risk, while 4 species are expected to be become extinct^77^. Therefore, this is a crucial time to reevaluate the coastal hazard impact on the SC coast through a new lens, which take into the account vulnerable cultural heritages, ecological resources, and social vulnerability, and -economic losses.

### Vulnerability indicators

#### Hydroclimate vulnerability indicators

**Figure 4:**
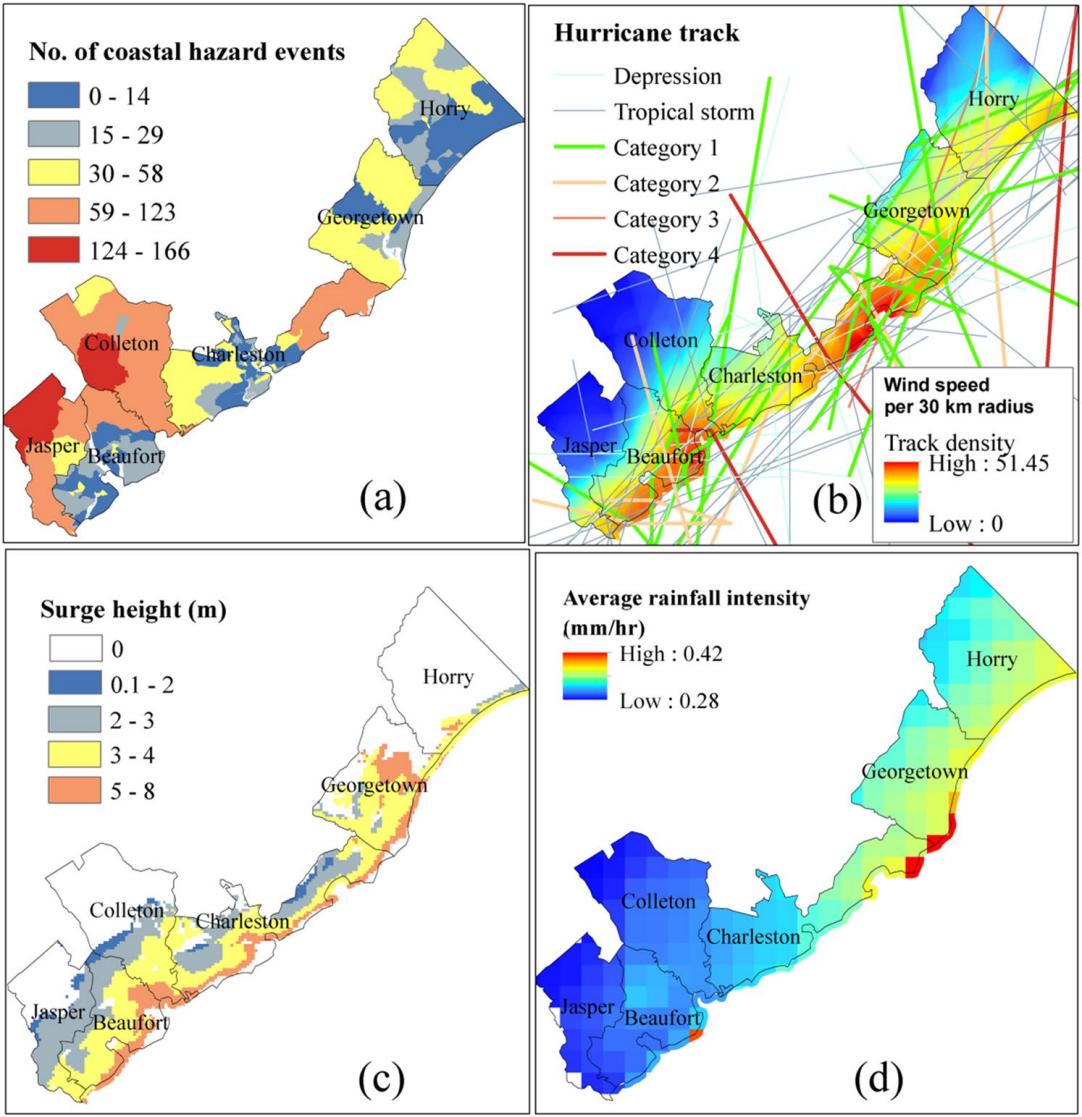
Hydroclimate vulnerability indicators (**a**) No. of coastal hazard events, (**b**) Hurricane track density, (**c**) Storm surge height, (**d**) Rainfall intensity.

The selected hydroclimate vulnerability factors are the number of coastal hazard events, hurricane track density, storm surge height, rainfall intensity, and SLR (Table 3). The statistics of total 4532 coastal hazard events during 1996–2019 in six coastal counties is utilized. Out of these hazard events most of them have been flood (69.9%), hurricane (22.34%), and then high tide events (4.25%). The average expected frequency of coastal hazard events in SC coast is 189 events per year, with 174 flood/hurricane events as the most anticipated hazard. Figure 4a shows spatial distribution of historic coastal hazard events in 226 census blocks. The property damage caused by historical hazard events in NWS records is estimated about US $23 million. Historically, Charleston County experiences the maximum numbers of annual coastal hazard incidents among coastal counties of SC, about 41 events per year, and followed by Jasper County with annual 23 coastal hazard events per year. The Georgetown County experiences the lowest number of coastal hazard events in the State with average of 21 events per year during 1996–2019. In this study, information of 124 hurricane events during 1851–2017 is derived in order to develop the cyclone track density map (Fig. 4b), using Eq. (1) (Silverman 1986).

10 $$Track \; density= \frac{\sum_{i=1}^{i=n}{L}_{i}\times {v}_{i}}{A},$$

where *L*_*i*_ is the length of the hurricane track (*i*), ${v}_{i}$ is the wind speed (km/h), and *A* is the radius of hurricane’s circle, which is assumed about 30 km in this study.

The total of fifty two historical hurricanes are classified into five hurricane category levels based on Saffir-Simpson scale which resulted in six Category-4, two Category-3, thirteen Category-2, and thirty one Category-1 hurricanes^78^. Most hurricanes have occurred during the hurricane season in SC, May–October. Here, the storm surge vulnerability in SC coast we employed the result from the National Hurricane Center (NHC) Sea, Lake and Overland Surges from Hurricanes (SLOSH) model. The SLOSH model system in U.S. coast is primarily developed for real time prediction of storm surge height, with the forecast accuracy within ± 20%^79^. The locations of peak surge in SLOSH model simulation are used here to develop the map for historical peak surge height and their spatial distribution (Fig. 4c).

Hurricanes have paramount role in bringing heavy rainfall to the coast and mid-state. The 0.1° resolution IMERG-Daily satellite-based precipitation product, is used to estimate the aerial precipitation intensity. Spatial precipitations produced by IMERG-Daily (final run) are retrieved from the Global Precipitation Measurement archive (Table 2). The average rainfall intensity of 0.14–0.22 mm/day, estimated during April 2014–Jan 2020, is used to develop the rainfall maps for the SC coast (Fig. 4d). Northern parts of Charleston, Georgetown, and Horry counties have experienced the highest rainfall accumulation during this period (Fig. 4d).

#### Physical vulnerability indicators

**Figure 5:**
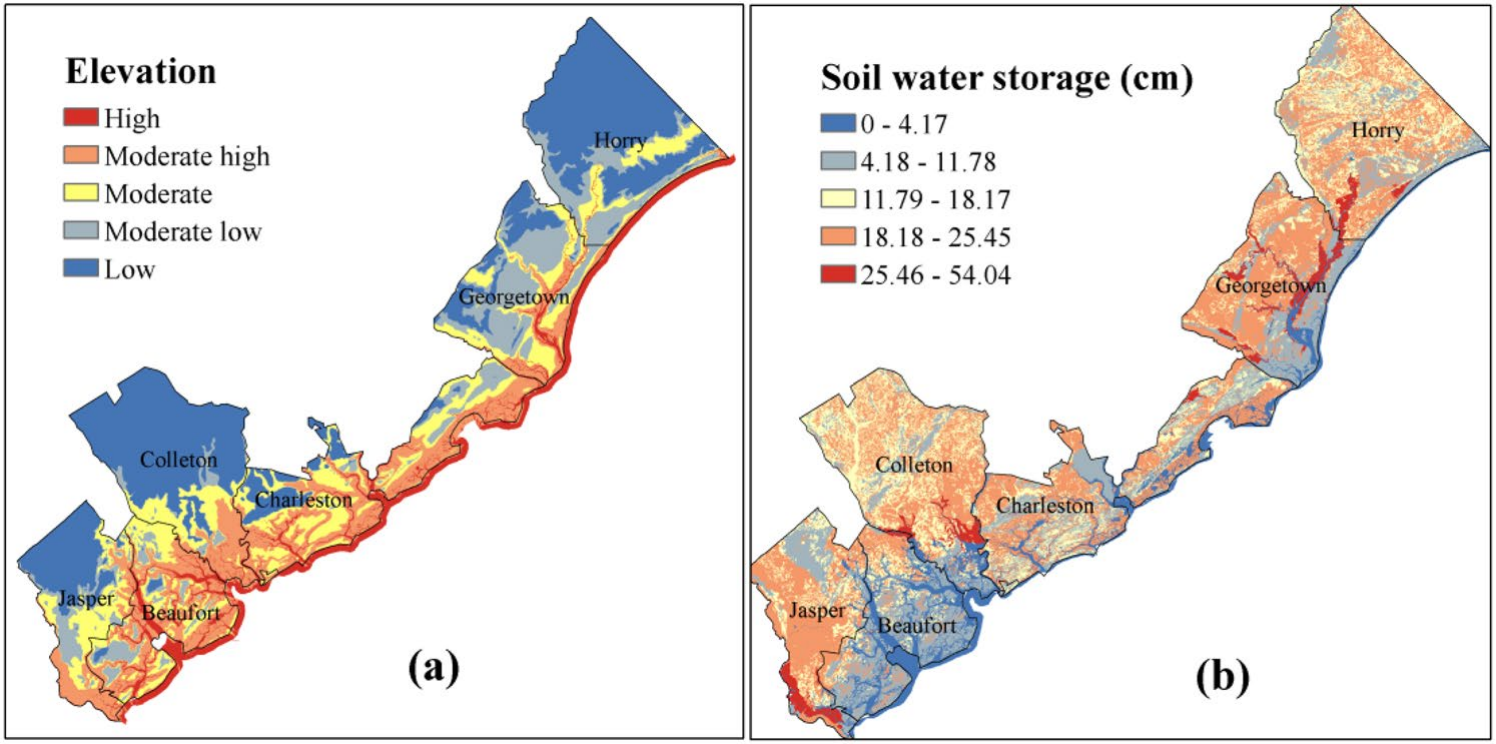
Physical vulnerability indicators (**a**) Elevation, (**b**) Available water storage in soil.

The physical factors refer to the natural geographical and built-in environment characteristics of the coastal system. Thus, including factors such as landuse (Fig. 3), average soil moisture content, elevation (Fig. 5), and distance from coastline are important to find hotspots for natural hazards. For example, areas with low elevation are highly susceptible to flooding caused by SLR and storm surge height. The distance is another inversely related factor with coastal hazard propagation. The farthest point of the study area is 109 km away from the shoreline, where the effect of coastal hazards are much reduced. Elevation maps as well as distance from coastline are used here to represent the physical risk of coastal flooding in land. Landuse is another key factor, which represents the human interventions on land, and in turn on the impact of hazards. USDA 30 m landuse map is obtained to analyze the land cover of the study area. The land cover map has 14 classes, water, open space, developed (low, medium and high intensity), barren land, deciduous forest, evergreen forest, mixed forest, shrub, herbaceous, hay/pasture, cultivated crops, woody wetlands, and emergent herbaceous wetlands (Fig. 3). About 30.91% of the area is covered by woody wetlands, followed by 14.34% of emergent herbaceous wetlands. The developed area only covers about 3.8% of the study area. The soil moisture content, contribute to the formation of flood, especially flash floods. The gSSURGO soil moisture data (Table 2) for the study area is engaged to get the available water storage (AWS) information (Fig. 5b).

#### Socio-economic vulnerability indicators

**Figure 6:**
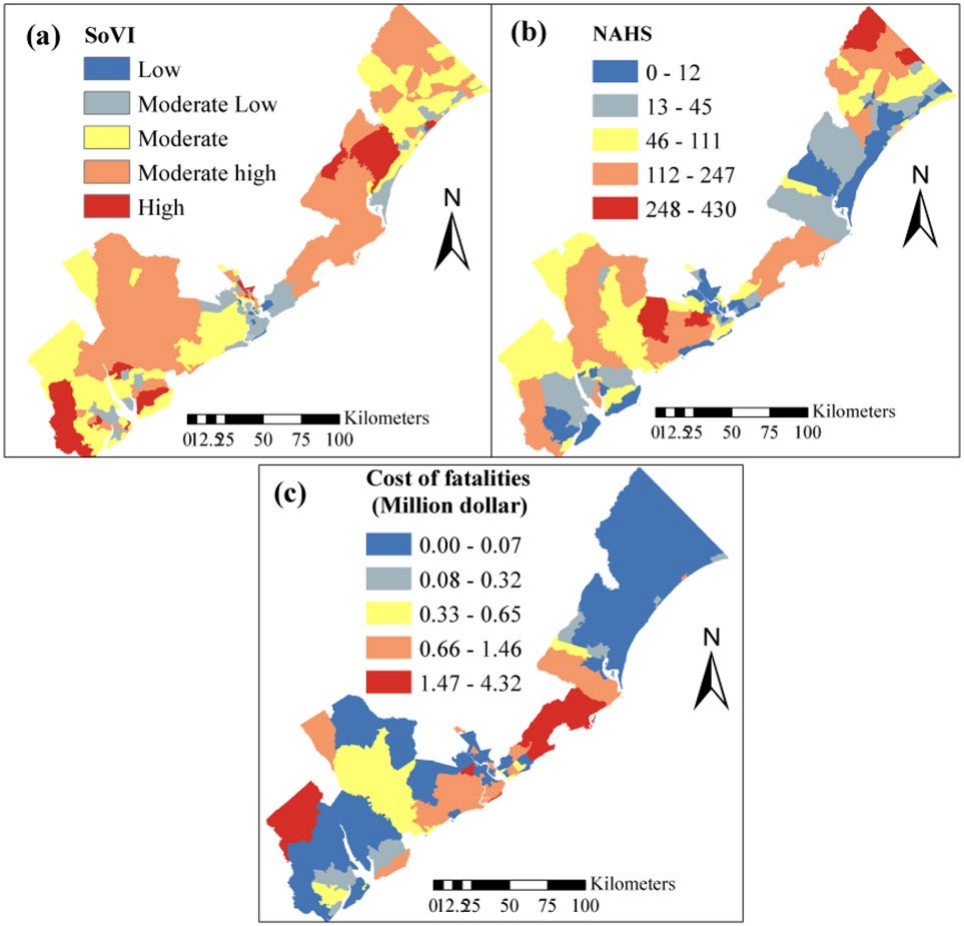
Socio-economic vulnerability indicators (**a**) Social Vulnerability index, (**b**) no. of Archeological and Historical structures (NHAS), (**c**) cost of fatalities.

Selected socio-economic vulnerability indicators, as a basis to estimate the adaptive capacity of system, are Social Vulnerability Index (SoVI, Fig. 6a) developed by Cutter & Emrich, (2017), Number of Historical and Archeological Structures (NHAS), and historical cost of fatalities for natural hazard events (Table 2). The Social Vulnerability Index (SoVI) and cost of fatalities caused by flood events are used as measures of societal resistance against coastal hazard (Fig. 6).

The culturally rich SC coast has a total of about 8749 Historical and Archeological structures (NHAS). Most of NHAS are in Horry County (3374) and the lowest NHAS are found in Georgetown (152). Charleston County has 2266 NHAS of which 80% of them are in the City of Charleston (Fig. 6b). The number of NHAS located within each of the census blocks is used as an indicator to measure the coastal hazard potential damage to historical and archeological sites and to tourism industry. Finally, the information related to the cost of fatalities is gained from NWS database for the historical storm events. This storm event database provides detailed description of natural hazard events with their geographic details, time, number of injuries and death directly and indirectly caused by the hazard, and estimated cost of property damage. The hazard events data in this study is represented at census tract level during 1996–2019 (Table 2). The cost of fatalities accounted here includes the property cost and the damage costs of crops (Fig. 6c).

#### Ecological vulnerability indicators

**Figure 7:**
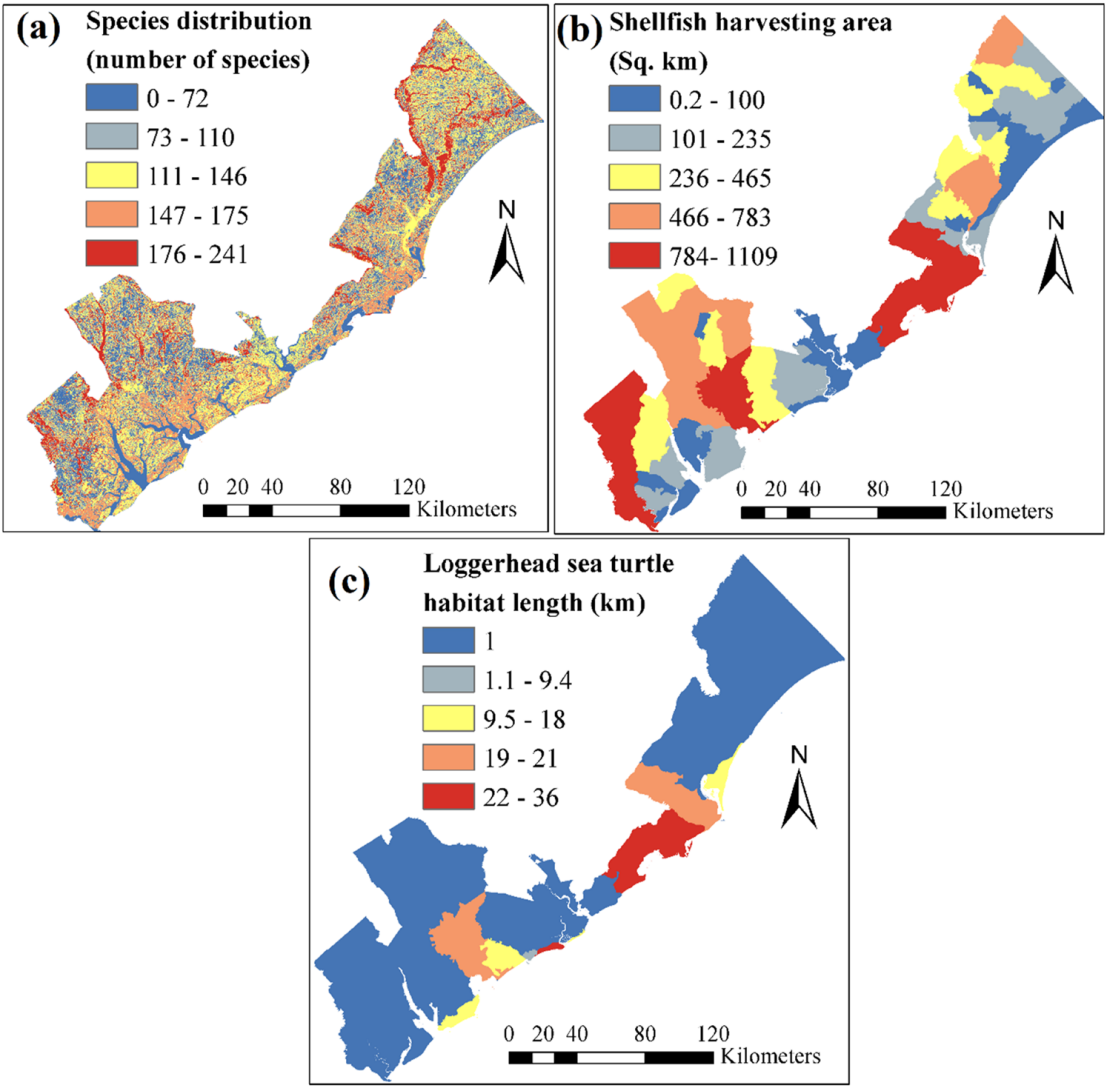
Ecological vulnerability indicators (**a**) Species richness, (**b**) Shellfish harvesting area, (**c**) Loggerhead sea turtle habitat.

The coastal ecosystem remains at the frontline of the coastal hazard. The species richness, area of turtle habitat and shellfish harvesting are among the main indicators for ecological vulnerability assessment of coastal systems (Fig. 7). The species distribution map developed by SC GAP project^57^ is used to represent the spatial distribution of species richness (Fig. 7a). Other two factors for ecological vulnerability analysis are chosen based on their ecological and economic importance in this region. The loggerhead sea turtle community is found endangered because of growing human activities near beaches. In the South East of U.S., about 6.5% nests of loggerhead sea turtles located in the SC coast^80^. Under 3 ft SLR projection, it is expected that these nests are completely vanished^77^. Many of shellfish harvesting areas are located in Charleston, Colleton, and Jasper Counties (Fig. 7b). Presence of numerous estuaries in SC coast has promoted shellfish harvesting in this region (Fig. 3). The estuaries are the host to about 75% of the total harvested shellfish in U.S., that contributes nearly $4.3 billion per year to country’s economy^81^.

#### Shoreline vulnerability indicators

**Figure 8:**
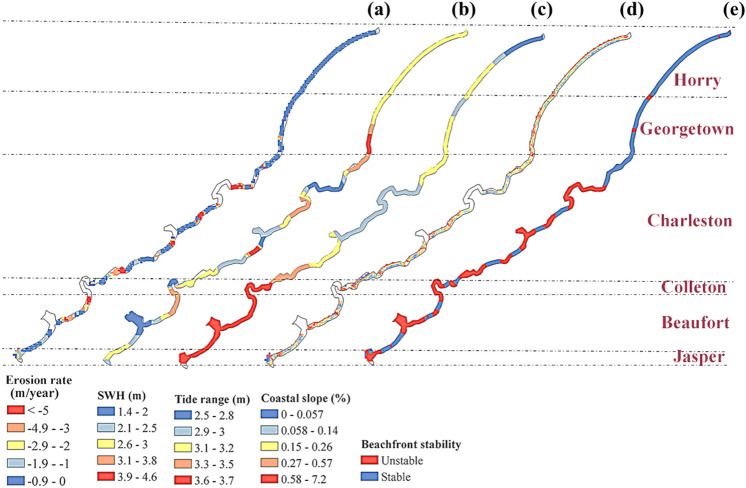
Shoreline vulnerability indicators of SC coast (**a**) Rate of shoreline change, (**b**) Significant wave height, (**c**) Tide range, (**d**) Coastal slope, (**e**) Beachfront stability.

The shorelines are located at a critical interface where dynamic interactions occur among ocean, atmosphere, and land. Five shoreline vulnerability indicators considered in this study, are the rate of shoreline change, significant wave height (SWH), tide range, coastal slope, and beachfront stability (Fig. 8). Information about the historical changes in shoreline are obtained from various databases, mainly USGS, for the period of 1852–2000 (Table 2). A total of 4921 transects or shoreline cross sections perpendicular to the beach are spaced at 50 m lengths to estimate the rate of shoreline changes.

The shoreline erosion rate is expected to be higher for Charleston and Beaufort Counties (Fig. 8a). The SWH is estimated for the 100 year return period (Fig. 8b). The tide range over a small zone does not show a large spatial variation (Fig. 8c). The tide range of above 50% exceedance probability, estimated by NOAA for 55 years (1955–2020), is utilized in this study (Table 1). Mean tide range throughout the year varies between 1.3–2.4 m in SC coast. Maximum tide range of 3.6–3.7 m was found near the Jasper and Beaufort Counties coast, which gradually decreases toward the Horry and Georgetown County (2.5–3.2 m) (Fig. 8c). The SC coastal region has a mild coastal slope, which can escalate the storm surge propagation by triggering high wave towards the onshore areas (Fig. 8d). The steeper slope in the nearshore areas prevents the propagation of surges by dissipating the storm surge energy. The SC coast is relatively flat, where a major portion of region has slope of less than 0.57%. Finally, beachfront stability is another important factor which implies to the shoreline vulnerability (Fig. 8e). The beachfront stability data are obtained from SCDHEC GIS database. The information about shoreline stability is classified into stable and instable beachfronts (Fig. 8e). The unstable beachfront can be found mainly in estuaries and tidal creeks (Fig. 8e).

## Results and discussions

In this section, first the results for vulnerability assessment of SC coastal region using MCDM with objective weighting method are presented. The uncertainty analysis of the spatial MCDM and heterogeneous spatial weighting to evaluate the rationality of obtained CVI over various spatial scales and extents are also discussed. Then, we apply the PPCA approach to form a new vulnerability index and develop vulnerability maps of SC coastal counties and compare the results with MCDM method. Finally, historical coastal hazard inundation maps and damage estimates are compiled to validate the CVIs obtained from both methods.

### Entropy-based weights for vulnerability factors

The weights are obtained from the entropy method within each vulnerability group (Table 4). The greater weight of the factor indicates a higher degree of influence on vulnerability. The cost of fatalities factor from socio-economic vulnerability group gained the highest weight among all the factors. Elevation, surge height, and turtle sites are dominant factors in each group of physical, hydroclimate and ecological vulnerability groups, respectively, based on entropic weighting. The rate of shoreline change has slightly greater weight in shoreline vulnerability group (Table 4).

**Table 4:** Weight of the factors from entropy method.

	Factors	Weight		Factors	Weight
Hydroclimate	No. of coastal hazard events	0.29	Ecological	Species richness	0.27
	Hurricane track density	0.22		Shellfish harvesting	0.32
	Surge height	0.31		Turtle sites	0.41
	Rainfall intensity	0.18	Shoreline	Rate of shoreline change	0.23
Physical	Curve number	0.21		Tide range	0.18
	Available soil water storage	0.24		Significant wave height	0.20
	Elevation	0.32		Coastal slope	0.19
	Distance from coast	0.23		Beachfront stability	0.21
Socio-Economic	SoVI	0.22			
	No. of historical and archeological structures (NHAS)	0.3			
	Cost of fatalities	0.48			

### Hydroclimate vulnerability

**Figure 9:**
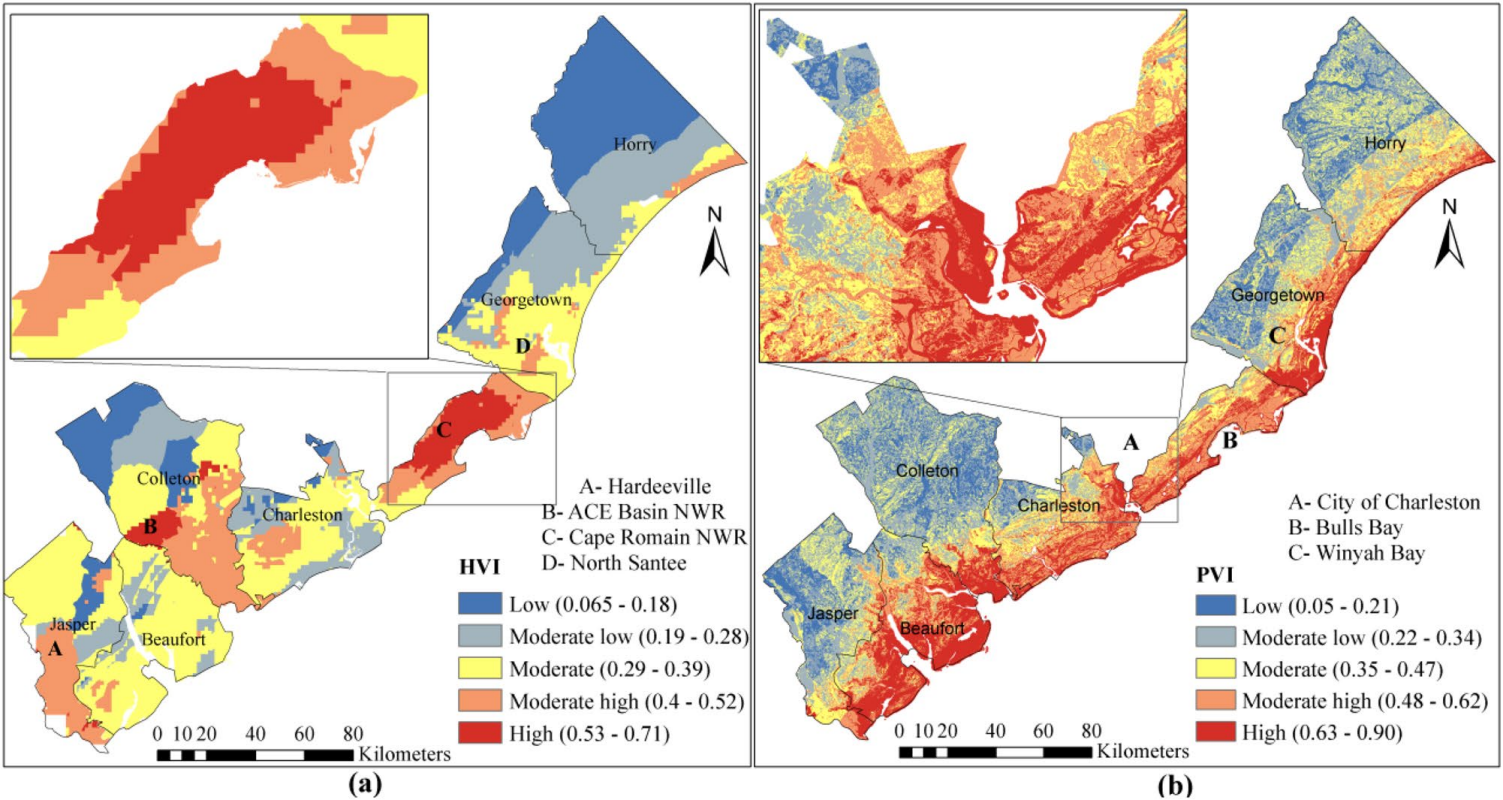
(**a**) Hydroclimate vulnerability, (**b**) physical vulnerability of SC coast.

A major portion (35%) of SC coast is prone to hydroclimate vulnerability. Based on the hydroclimate vulnerability map (Fig. 9a) 22%, 23%, 35%, 15% and 5% of SC coastal region has low, moderate low, moderate, moderate high and high vulnerability classes, respectively. The Charleston County is found as the most vulnerable region based on HVI. The Cape Romain NWR of Charleston County (shown on Fig. 9a) is located at high hydroclimate vulnerability, mainly due to intense hurricane activities in that region. The Charleston County has experienced 16 hurricanes (the highest number of hurricanes), followed by Beaufort County with 10 Historical hurricanes during 1996–2018. Historical surge height records had been recorded at three places of SC coast, estuaries of Beaufort County, Winyah Bay, and Charleston harbor with the surge height of about 5–8 m. Presence of numerous tidal rivers, such as Edisto River, Salkahatchie River, in Beaufort County expedites the surge propagation toward inland. Many coastal hazard events were occurred at ACE Basin NWR, which resulted in high hydroclimate vulnerability in this area (Fig. 9a). Southern part of Colleton and Jasper Counties and North Santee of the Georgetown County are moderately vulnerable (Fig. 9a). The Horry County has a low vulnerability, mainly due to its low number of historical hurricanes and coastal hazard events. The storm surge cannot penetrate further inland in Horry County as there are a smaller number of large estuaries in this county, compared to other counties.

### Physical vulnerability

The coastal areas near the Atlantic coast are recognized as highly vulnerable regions, as those areas are highly susceptible to be inundated under projected SLR level of 0.1–0.3 m by 2050. The Beaufort is the most vulnerable county with spatial average of PVI of 0.58, followed by Charleston with PVI of 0.52. Colleton is the least vulnerable County (Fig. 9b). The PVI results show that 16%, 30%, 23%, 15%, and 17% of SC coast has low, moderate low, moderate, moderate high, and high physical vulnerability, respectively. The shoreline length of a county shows a direct correlation with PVI. For instance, Jasper County has shoreline length of 9.64 km and spatial average of PVI of 0.29. This is also true for Colleton County with a small shoreline length (20 km) which has PVI of 0.32 (Fig. 9b). The percentage of developed land and impervious area at coastal urban centers, such as the city of Charleston, is another important factor that contributes to a higher degree of vulnerability.

The low-lying lands adjacent to the coast are more susceptible to natural hazards such as flood. Low elevation of the land, e.g. Charleston Peninsula with about 80% area has elevation below 4 m, is another key factor in vulnerability assessment. A major portion of the Charleston City is located 1–1.5 m higher above the regular high tide line. These pose serious challenges for urban development and planning and sustaining the cultural resources, public and private properties from potential flooding caused by storm surge and SLR. Moreover, the high tides cause backflows in coastal drainage system, such as those at the adjacent Ashley and Cooper Rivers, and in turn more frequent nuisance flooding in this region. In 2016 and 2017 the tidal flood days was recorded 50 and 46 for City of the Charleston^71^. U.S. Army Corps of Engineers proposed a $1.8 billion coastal management plan for Charleston peninsula to increase the adapting capacity of the City and alleviating the region’s physical vulnerability by developing new structural measures, such as storm surge barriers, breakwater or wave attenuation structures, deployable floodwall, levees, and elevate the roads, bridges and building. A coastal ecosystem consists of various components including saltmarshes, emergent marshes, coastal forests, coral reefs, mangrove forests, aquatic vegetation in coastal wetlands, etc. These elements can act as shoreline measures to reduce the risk of coastal hazards. For instance, saltmarsh reduces the adverse effect of salt intrusion due to SLR^82^, decreases the storm surge and wave height through increasing the flow resistance of water, and reduces the surge wave propagation speed^83^. Given the growing interests in quantifying the ecological factors and their interaction with the socio-economic factors, a few number of studies tended to perform bi-variate coastal vulnerability analysis (e.g., Refs.^43,53^).

### Socio-economic vulnerability

The adaptive capacity of a system can be measured with respect to technological and structural measures, such as early warning systems, emergency services, and long-term control planning strategies. For example, lifting the structures elevation above 1% annual flood exceedance probability, relocating of the valuable and in-danger building out of the floodplain, and land use regulations can significantly reduce the coastal hazard damage. In addition to the structural measures, the nonstructural measures, such as level of education, public health, income, etc., contribute to the adaptive capacity of a region.

**Figure 10:**
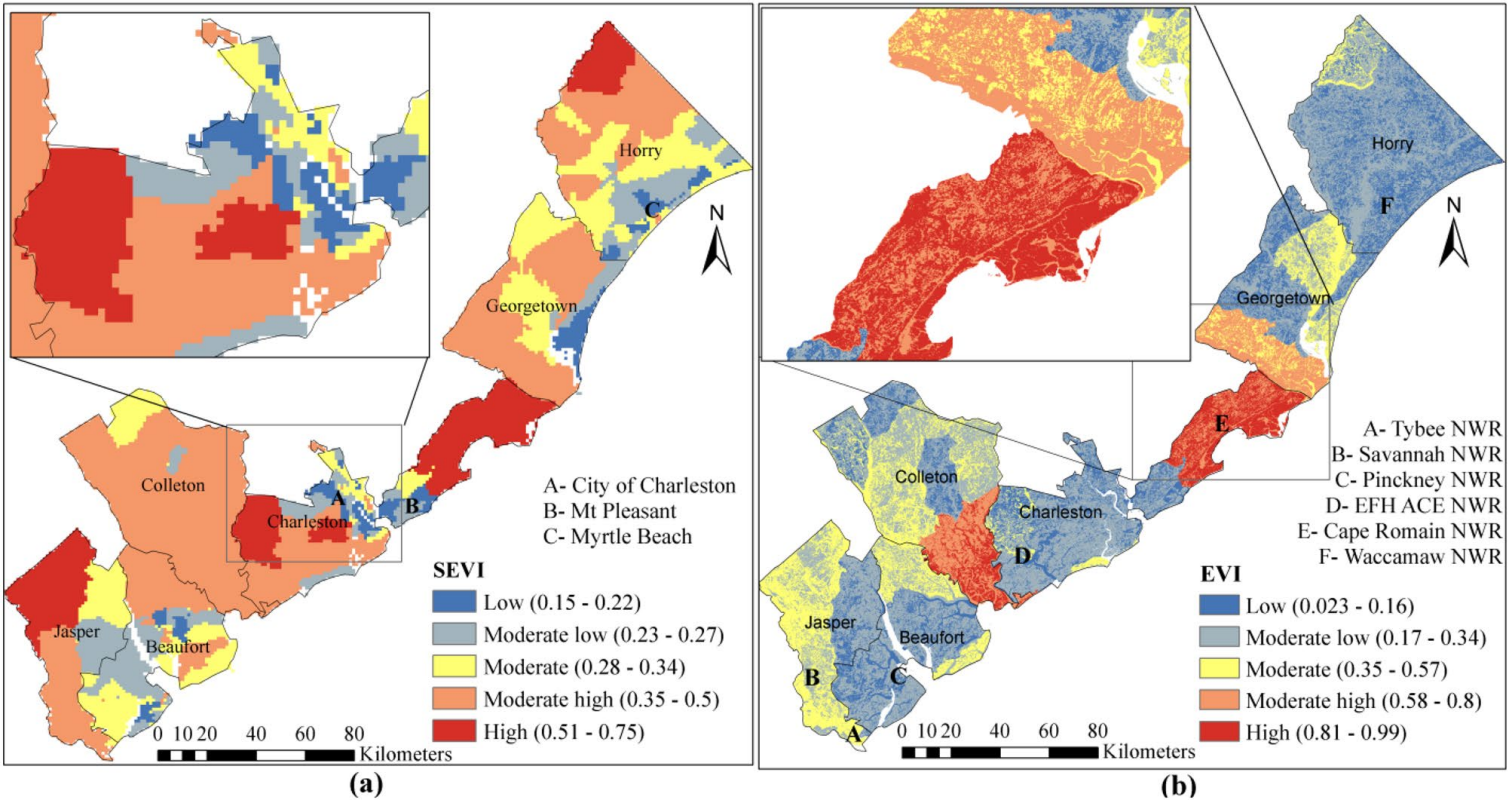
(**a**) Socio-economic vulnerability and (**b**) Ecological Vulnerability of SC coast.

A low to moderate socio-economic vulnerability has been discerned at most populated areas along SC coast, such as the cities of Charleston, Mount Pleasant, Myrtle Beach and Beaufort County (Fig. 10a). A great difference has been seen in adaptive capacity of coastal hazard between urban areas and countryside in SC (Fig. 10a). The north-western and south-eastern part of the Charleston County were found as moderately high and highly vulnerable regions. That is the part of the county where minority communities and scattered disadvantaged communities are located. The result of SEVI estimation shows that 4%, 12%, 20%, 48%, and 16% of the study area is classified as low, moderate low, moderate, moderate high, and high socio-economic vulnerability. Colleton has the highest degree of socio-economic vulnerability (Fig. 10a).

### Ecological vulnerability

Results from ecological vulnerability estimation show that 22%, 46%, 18%, 8%, and 6% of the SC coastal region have low, moderate low, moderate, moderate high, and high ecological vulnerability (Fig. 10b). Six NWR are located in coastal region of SC. Among them, only the largest one, Cape Romain NWR, holds a high ecological vulnerability (Fig. 10b), rest of them are located at low to moderate classes. Coastal recovery and management plans in the future should focus minimizing the high vulnerability of Cape Romain NWR. In addition to ecological vulnerability, the physical vulnerability of this NWR is showing that the Cape Romain NWR is located at high physical vulnerability region as well.

Natural protective measures for coastal area acts as a buffer against the coastal hazard by reducing wind erosion, wave attenuation and tidal inundation. Promoting the marsh wetlands can potentially slow down the storm surge speed. Subsequently, it is expected that preserving current coastal wetlands can protect the inland areas by reducing the SLR effects over time. Moreover, the erosion prone shorelines can be restored by planting vegetation. For instance, the oyster reef toe can hold the sediments to reduce the erosion.

### Shoreline vulnerability

**Figure 11:**
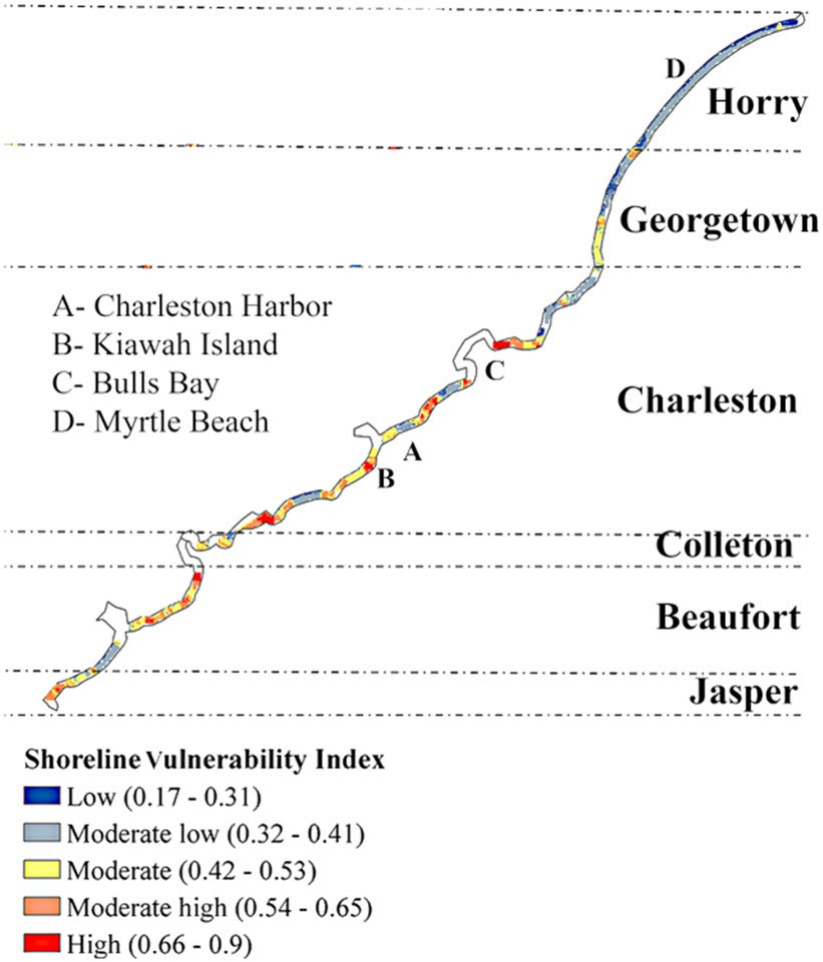
Shoreline vulnerability of SC coast.

Shoreline vulnerability in SC is broadly classified under moderate low vulnerability class which makes up about 41% of the entire shoreline (Fig. 11). About 7.4% of the SC coastline is classified under high vulnerability, most of which are located in the Charleston and Beaufort Counties. Rest of the shoreline can be described as low vulnerable (about 10%), moderate vulnerable (about 22%), and moderate high (about 20%) (Fig. 11). The rate of shoreline change is the dominant factor among the shoreline vulnerability indicators since this factor carries the majority (0.31 out of 1) of weight for shoreline vulnerability estimation. Myrtle Beach has the lowest shoreline with relatively better coastal characteristics, such as stable beachfront, low tide range, moderate SWH. Charleston harbor and Kiawah Island (Fig. 11) are found highly vulnerable due to their flat coastal slope and unstable beachfront. It is expected that, the tourism industries take advantages of favorable shoreline coastal characteristics at Georgetown and Horry County in the future.

Several protection measures can withstand against shoreline erosion. Intertidal sandy shoals are resilient features which perform well against shoreline erosion and SLR. Several observations and modeling (e.g., Refs.^84,85^) suggested that the sandy shoal can achieve stable morphodynamic form at coastal intertidal wetlands and creeks by balancing multiple coastal forcing including sediment supply, tidal action and wave erosion. Intertidal mud and sandy shoals at the seaward shoal edge gradually propagates landwards with SLR and as a results shoreline can achieve a long-term equilibrium^84^. Numerous mudflats in the intertidal creeks, wetlands and estuaries of SC coast should remain unaltered by human intervention to make the SC coast more SLR resilient. Deepening project in Charleston harbor can accumulate the dredged sediment to elevate the adjacent mudflats as a nonstructural and cost-effective solution against shoreline erosion.

### Sensitivity analysis

After estimating different vulnerabilities, they need to be integrated to form the coastal vulnerability index. Here, a sensitivity analysis has been done on spatial weights of indices to evaluate the sensitivity of obtained coastal vulnerability across spatial scales and extents.

**Figure 12:**
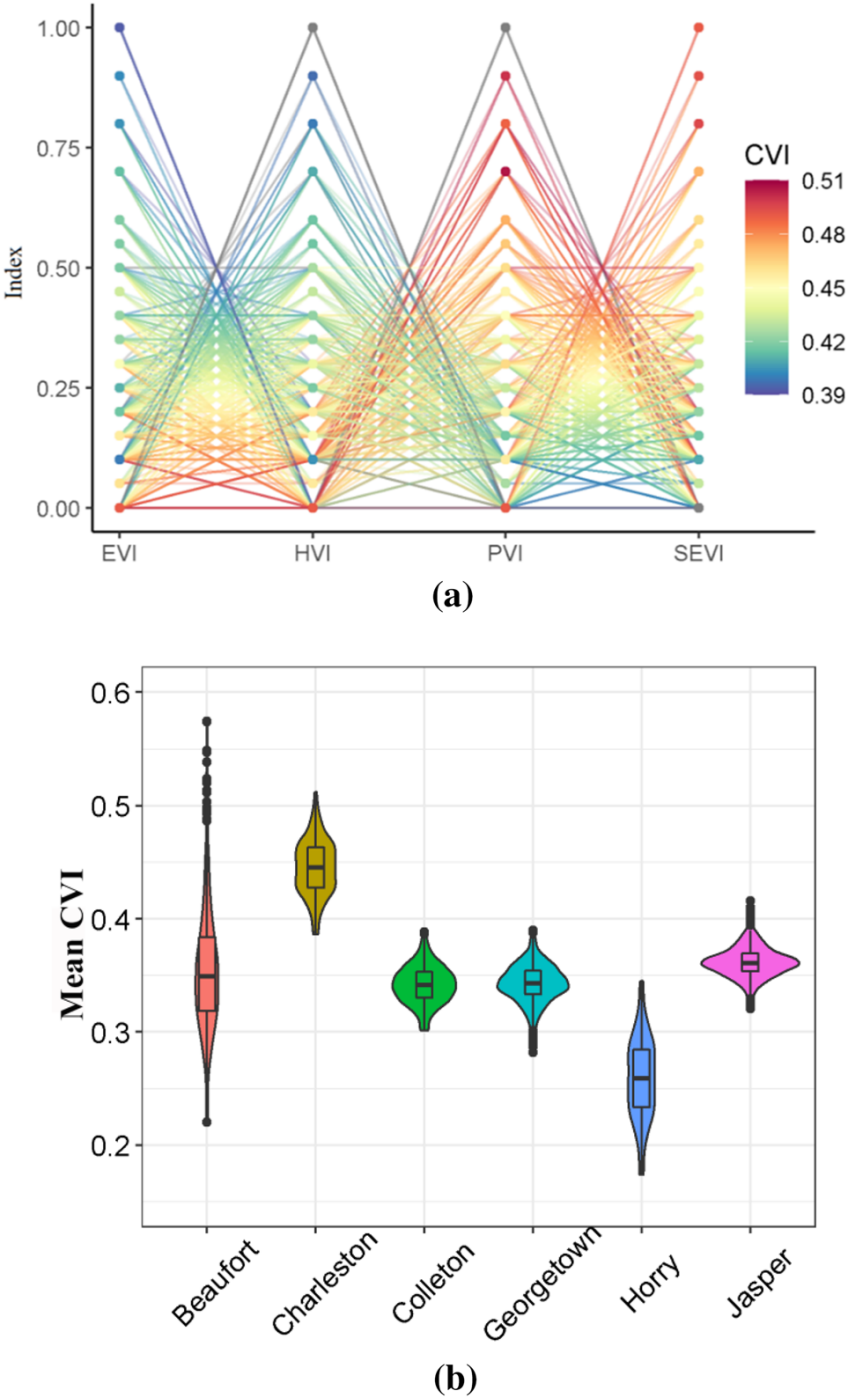
(**a**) Parallel coordinate plot of the vulnerability group’s weight drawn for the Charleston County. (**b**) Violin plot showing the mean CVI of different county varies with changing weights of the vulnerability groups.

A parallel coordinate plot of the Charleston County, as an example, is shown in Fig. 12a. A total combination of 730 weights are simulated, noting that sum of the weights should be always equal to 1. Each line of the parallel coordinate plot represents a combination of weights of four vulnerability groups (a, b, c, d in Eq. 8), while the color of the line indicates the mean CVI over the county. The weight of each group varies in a range of 0 to1. For Charleston County, the final CVI is mostly sensitive to the changes in SEVI and PVI weights. The weights for hydroclimate and ecological vulnerability indices have less impact on the estimation of CVI, and as a result more attention should be taken for estimating the SEVI and PVI in this county.

The sensitivity analysis has been done separately for each county and the probability of changes in CVI is shown in Fig. 12b for all the counties. The histogram and bar plots are also shown within the violin plot (Fig. 12b). The Beaufort County has the highest rate of changes in CVI (0.21–0.58), and thus more sensitive to the selection of weights for different vulnerability indices among these counties (Fig. 12b). The Colleton and Georgetown are less sensitive to the changes of vulnerability group’s weights. Similarly, there is a little impact of changing the weights of vulnerability groups on final CVI for the Jasper County. Figure 12b shows that, regardless of the vulnerability indices weights, the Charleston County has the highest coastal vulnerability index (0.4–0.5). On the other hand, the Horry County is the less vulnerable than other counties. The 50th percentile weights of the vulnerability groups ranges between 0.2 and 0.3 for the these counties, except the Beaufort. For the 50th percentile weights, the HVI has the highest importance in Beaufort County), while Charleston County is more sensitive to SEVI; Colleton County is more sensitive to EVI; Horry County is sensitive to PVI (Table 5). The Georgetown and Horry are less sensitive to SEVI, while other counties are highly sensitive to the changes in SEVI weight (Table 5).

**Table 5:** List of weights of the vulnerability groups for 50th and 90th percentile CVI.

Index	Jasper		Beaufort		Colleton		Charleston		Georgetown		Horry	
	^a^Q_50_	^b^Q_90_	Q_50_	Q_90_	Q_50_	Q_90_	Q_50_	Q_90_	Q_50_	Q_90_	Q_50_	Q_90_
EVI	0.2	0.3	0.2	0.3	0.25	0.45	0.3	0.1	0.3	0.4	0.25	0.1
HVI	0.2	0.15	0.45	0.1	0.2	0.15	0.2	0.1	0.2	0.1	0.25	0.1
PVI	0.3	0.1	0.2	0.3	0.3	0.1	0.25	0.3	0.2	0.4	0.25	0.7
SEVI	0.3	0.45	0.15	0.3	0.25	0.3	0.25	0.5	0.3	0.1	0.25	0.1

^a^50th percentile of weight in sensitivity analysis.

^b^90th percentile of weight in sensitivity analysis.

### CVI-50 and CVI-90 vulnerability maps

**Figure 13:**
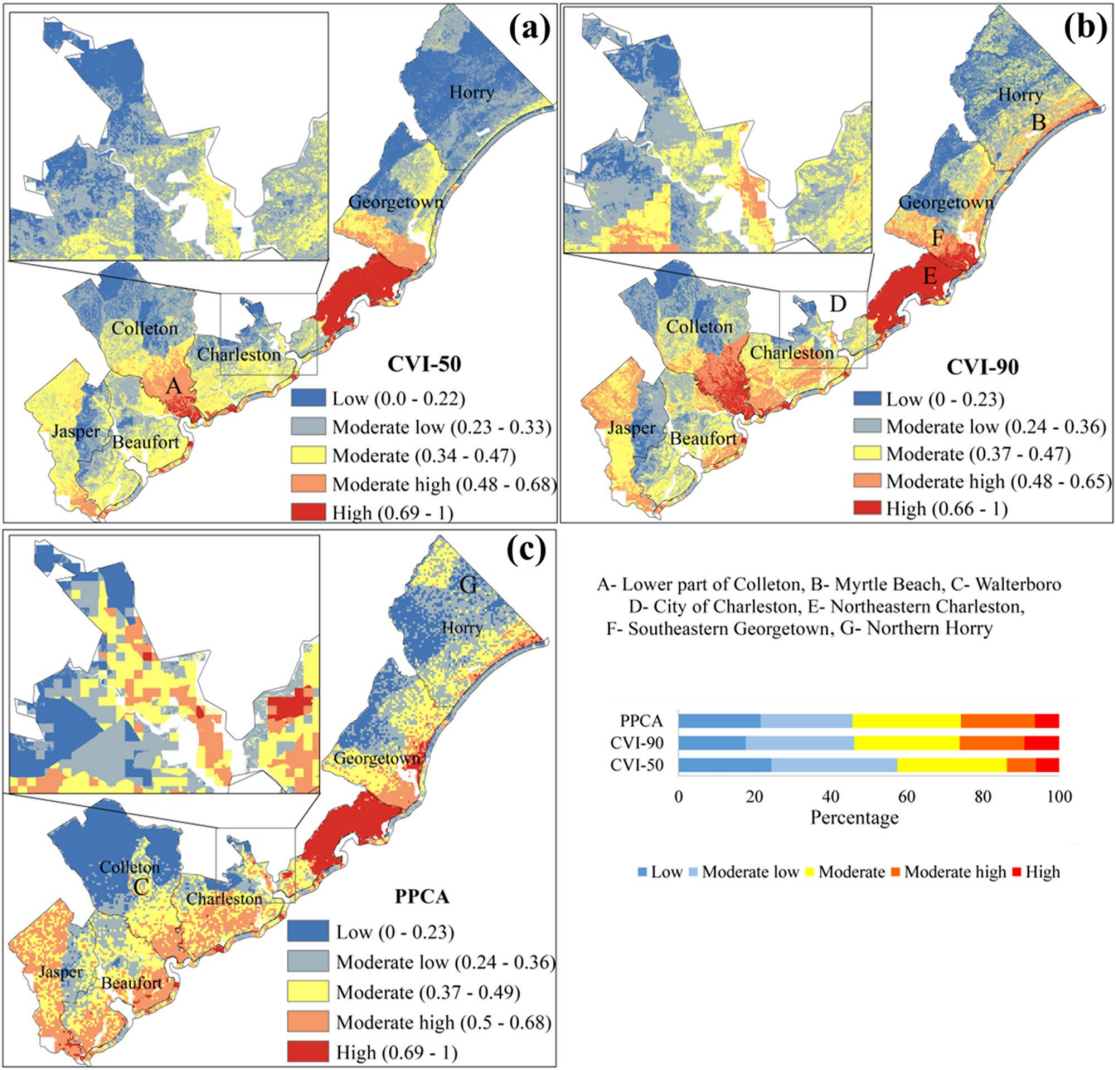
Coastal vulnerability map estimated by (**a**) CVI-50, (**b**) CVI-90 and (**c**) PPCA method.

The generated vulnerability maps based on CVI-50 and CVI-90 weights from Table 5 are shown in Fig. 13a,b. The CVI-90 method focusses more on sensitive vulnerability index to assign CVI weights, whereas the CVI-50 resembles an average combination of all vulnerabilities. The northeastern part of the Charleston County is identified as the most vulnerable place in SC coast. This area has been identified as a hotspot in hydroclimate, physical, socio-economic and ecological vulnerability assessments. A major portion of Charleston County was marked as moderate to highly vulnerable. The high-level of adaptive capacity in City of Charleston and adjacent suburbs resulted in low and moderate level of vulnerability based on CVI-50 method. However, the level of vulnerability under CVI-90 is higher than the CVI-50 (Fig. 13a,b) as the Charleston County is more sensitive to both SEVI and PVI (Table 5). The Myrtle Beach (Fig. 13b) appears as a moderately high vulnerable area under CVI-90 map. This part of Horry County experiences severe storm surges and landfalling hurricane in recent years. The enduring storm surges from the adjacent North Carolina coasts also affect the Myrtle Beach. The southeastern part of Georgetown County appears as moderately high to highly vulnerable class (Fig. 13b), mainly due to moderate high EVI and SEVI. Similarly, lower part of Colleton demonstrates a high vulnerability with high EVI and moderate high SEVI (Fig. 13a,b). The lower portion of Colleton is found vulnerable to ecological factors (Fig. 13a,b). The shellfish harvesting area, turtle sites and species richness are abundant in this part of SC. The Beaufort County largely appears as a moderate low to moderate vulnerability class based on CVI-50, however, some certain parts of the county turn into moderate high in CVI-90 map (Fig. 13a,b). The reason for this is the high PVI and moderate HVI of these locations. Jasper County appears as moderately vulnerable under both CVI-50 and CVI-90 maps. Socio-economic vulnerability of moderate high to high level has a noticeable effect on the vulnerability of Jasper County. The variability of CVIs, according to assignment of weights, in SC coast demands for adaptive and robust development strategies and climate change adaption plans with catering vulnerable hotspots associated with socio-economic and ecosystem to withstand the coastal hazards.

### Vulnerability map based on PPCA

**Figure 14:**
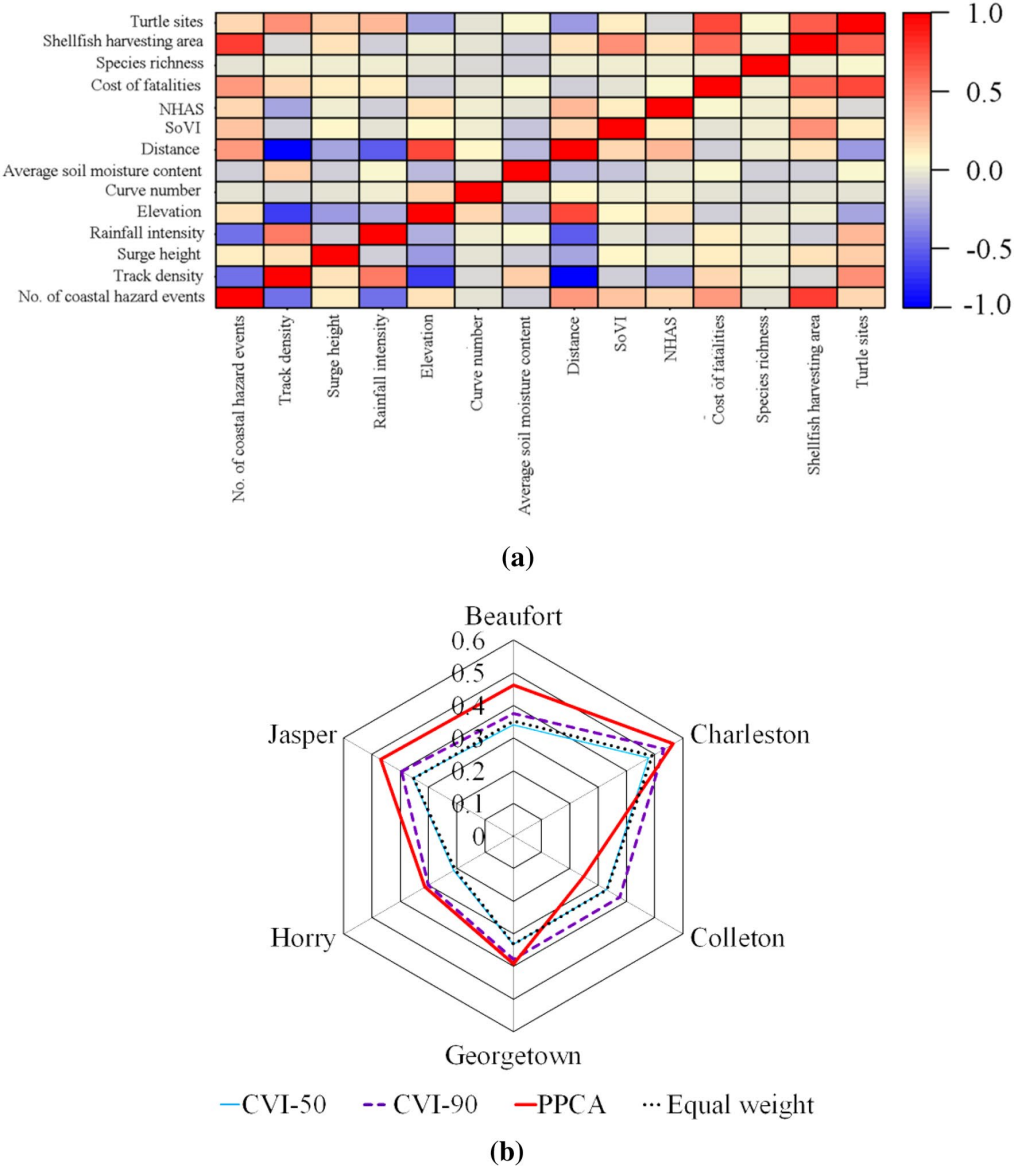
(**a**) Spearman correlation matrix of the vulnerability indicators. (**b**) Variation of mean CVI in six coastal counties with different methods.

The correlation among the selected factors for PPCA is shown in Fig. 14a, where a strong correlation is found between elevation and distance with track density. Moreover, five PCs are selected for the PPCA analysis (Table 6). The results show that these 5 PCs in PPCA have about 80.2% of the cumulative variance from the original factors. The first component of PPCA (PC1) maintains the largest portion of the cumulative variance, 46.13%. Hurricane track density, distance and elevation have higher loadings and are positively correlated with PC1 (Table 6). Average soil water storage and elevation vulnerability are positively correlated with the second component (PC2) of PPCA, while the PC2 maintains 10.32% of the total variance. The third component (PC3) is positively corelated with socio economic and ecological vulnerability indicators (Table 6). It can be seen that (Table 6) the cost of fatalities increased with the no. of coastal hazard events, area of shellfish harvesting, species richness, NHAS and turtle sites in PC3. The fourth component, PC4 with 8.15% of variance, has positive correlation with storm surge height and SoVI. The fifth component, PC5 with 6.35% of variance, has only one positive correlation in it, elevation factor, and the rest of other factors demonstrate negative correlations.

**Table 6:** Results of factors loading after varimax rotation.

Factors	PC1	PC2	PC3	PC4	PC5
No. of coastal hazard events			0.141		
Storm surge height			− 0.122	0.951	− 0.114
Track density	0.552				− 0.150
Rainfall intensity	0.145				− 0.264
Curve number					− 0.346
Elevation	0.184	0.369	0.697	− 0.177	0.333
Average water storage of soil		0.902	− 0.241		
Distance	0.749				
SoVI	− 0.107			0.131	− 0.702
Cost of fatalities			0.104		
NHAS	− 0.158		0.297		
Species richness			0.299		− 0.312
Area of shellfish harvesting	− 0.153	− 0.119	0.451		− 0.141
Turtle sites			0.2		
Explained variance (%)	46.13	10.32	9.25	8.15	6.35
Cumulative variance (%)	46.13	56.45	65.7	73.85	80.2

In PPCA method, the vulnerability of any specific place cannot be directly explained by original classification of the vulnerability groups and factors, instead this should be done by analyzing the PCs. The factor loading can be used to bridge between the PCs and original vulnerability indicators. The PC1 has the largest weight in PPCA method, with high factor loadings associated with physical and hydroclimate factors, in particular distance, track density, elevation and rainfall intensity. The place which is found highly vulnerable under the criteria of distance and high track density is therefore expected to be identified as vulnerable in PPCA based vulnerability map as well. Larger hurricane activities and proximity to the coast make the northeastern part of Charleston highly vulnerable.

The spatial pattern of PPCA based CVI is very similar to CVI-90 with few exceptions. The elevation factor notably influences CVI as seen in previous sections. Here, elevation has positive correlation and high loadings for all PCs is found except PC4. Jasper County has dominance of moderate vulnerable pixels under CVI-90 method, but this county is changed to mainly moderate high vulnerability class by PPCA method. Similarly, Beaufort County seemed to be dominantly moderate low and moderate vulnerable pixels under CVI-90 method, but PPCA based CVI shows this county tends to be more moderately highly vulnerable. It is seen that Charleston County is identified as the most vulnerable County in SC coast regardless the method used for vulnerability assessment.

The Colleton County has the lowest CVI among other Counties. So, one may argue that the underlying reason is its positive correlation with factors associated environmental vulnerability Table 4 confirms that the Colleton is mostly sensitive to the environmental vulnerability. Also, final PPCA map is less influenced by PC3 factors, where mostly environmental vulnerability -related factors exist.

Vulnerability of Beaufort County was found higher in PPCA method than CVI-50 and CVI-90 methods. This can be due to of the effect of higher importance of hurricane track density factor. Similarly, the City of Charleston was marked as moderately to highly vulnerable area due to its low elevation and high hurricane track density which have higher prioritization in PPCA method (Fig. 13c). Northern part of Horry shows notable increase in vulnerability compared to MCDM methods, because this region has higher SoVI influence and stronger correlation from PC4 (Fig. 13). Although PPCA method recognizes this part as moderate vulnerable, CVI-90 disregards the effect of SoVI, since the Horry County is sensitive to physical vulnerability.

The estimated CVIs by all the three methods is shown in spider plot for each of six counties (Fig. 14b). The CVI was also estimated based on an additional scenario, using equal weights for all 14 vulnerability factors (excluding shoreline class) (Fig. 14b). The equal weight method is a conventional way of estimating CVI and has been used previously in several studies^31,32^. CVI-50 and Equal weights method provide quite identical CVIs for all six counties. The prominent difference between the CVI-50 and CVI-90 is that the CVI-90 reflects the extreme vulnerability condition by increasing the weights of most sensitive vulnerability groups, while the CVI-50 tends to depict the normal conditions. PPCA method provides the highest CVI among all three methods except in case of Colleton County.

### Validation of vulnerability maps

The satellite images have been used to derive historical flood maps and cost of fatalities to validate the CVIs. A binary classification has been used to classify the CVI results into vulnerable vs. non-vulnerable at a pixel level using the natural break classification method. The vulnerability prediction results in CVI maps were judged by true positive, representing observed vulnerable pixels correctly classified as vulnerable, and true negative, representing true non-vulnerable pixels correctly classified as non-vulnerable. False positive and false negative samples are those incorrectly classified pixels as vulnerable (positive) and non-vulnerable (negative). The validation has been done by taking 368 random samples (pixels) from flood map and 53 places for estimating the damage costs. Table 7 shows the results of validation process for two separated overall categories of natural risk (represented by flood) and socio-economic damage (represented by cost of fatalities). A pixel-wise comparison of the CVI products showed that PPCA has a closer match with the post-hazard flood maps (Table 7). It can be concluded from Table 7 that the CVI-50 may result in underestimation of actual degree of vulnerability, as less number of pixels are detected, less projected true positives compared to two other methods in both natural risk and socio-economic damage. PPCA has better accuracy to project the risk of natural hazards and less accuracy to explain the socio-economic damages. As seen from Table 6, PPCA is less sensitive to socio-economic factors, lower weights for associated components of PC3 and PC4. CVI-90 and PPCA methods improve the performance of projecting the true positive samples in natural risks, but at the same time these methods slightly underestimate the true negative samples of socio-economic vulnerability.

**Table 7:** CVI validation with flood and socio-economic data.

Validation data type	Method	True positive	False positive	True negative	False negative
Flood	CVI-50	169	68	60	71
	CVI-90	187	70	58	53
	PPCA	195	81	47	45
Socio-economic Damage	CVI-50	6	15	31	1
	CVI-90	7	22	24	0
	PPCA	5	26	20	2

## Discussion

Traditional (top-down) coastal vulnerability studies focus mostly on biophysical factors and less on socio-environmental factors (bottom up). This is an important gap as the combination of both will often result in more damage when a flooding event occurs making the coastal system more vulnerable. This study investigates the interrelationship of socio-environmental and biophysical factors tying together both top-down and bottom-up factors to develop an integrated index. For this purpose, a multi-variate approach is used to form an informed integral CVI. This index is composed of a wide range of physical, hydroclimate, socio-economic, ecological, and shoreline factors of SC.

The most important task in fine scale vulnerability mapping and assessment is to deal with big geospatial datasets. For example, a 30 m resolution CVI map has approximately 69 million pixels for each of the involved vulnerability factors, which requires considerable computational power and analysis time. Thus, the improved MCDM and PPCA for geospatial data analysis framework can provide better understanding on fine scale CVI development, and a complete perspective of vulnerability assessment. Here, GIS and remote sensing techniques along with data driven approaches are used to develop the CVI. In addition, some of the presented methods for estimating individual vulnerability factors will require up to date and more advanced quantification in future. For instance, this study uses LRR methods’ from USGS National Assessment of Shoreline Change Project^58^ for obtaining the long term (1851–2000) rate of shoreline change. However, LRR is sensitive to data clustering over times and underestimates non-linear shoreline erosion processes that may affect the rate of shoreline change.

Conventional MCDMs, such as the Analytical Hierarchy Process, rely on expert or user experience-based judgements to identify importance of vulnerability factors. These techniques are prone to biases that arise from individuals’ judgements. Our study also found that using equal weights, i.e., similar level of importance, among the vulnerability groups creates discrepancies in CVI estimation as coastal vulnerability does not necessarily have similar sensitivity throughout the study area to all the factors. To address this issue, the sensitivity analysis of CVI has been performed with regards to the vulnerability factors. To represent the sensitivity of coastal vulnerability to the uncertainty associated with weighting the involved factors, two versions of CVI maps, CVI-50 and CVI-90, based on average and extreme values of the factors are developed. Moreover, Probabilistic Principal Component Analysis (PPCA) is used to better represent the hidden relationships and increase the interpretability of CVI factors. Overall, PPCA is a useful approach in approximating the missing values of spatial factors.

Finally, to validate the level of precision of our vulnerability maps, observed NWS cost of fatalities records and Sentinel-1 flood inventory maps are used. Comparing results shows that the MCDM-based CVI-90 and PPCA outperform the CVI-50, which represents the traditional vulnerability assessment. The proposed methodology and findings of this study contribute to the state-of-the-art coastal vulnerability assessment and provide accessible, reproducible, and transferable methods and tools so that similar vulnerability assessments may be performed for other type of hazards and at different locations**.**

## Conclusions

Over the past decades the coastal vulnerability analysis has been confined either biophysical or socio-economic dimensions. The need for an integrated vulnerability assessment is most compelling solution by combining biophysical, ecological, and socioeconomic vulnerability dimensions in broad sense. Additionally, the dominant factors in shoreline considering all oceanographic forces can reveal a complete picture of coastal vulnerability. Weighting of the vulnerability factors is an effective descriptor of the importance of a vulnerability indicator. However, the subjective weights elicited from expert judgment are prone to be biased by human perception. This leads to search for more appropriate and heterogeneous spatial weighting technique in MCDM. This study highlights important features which should be taken into serious consideration in future vulnerability assessment studies:

1. Results of this study indicates the great performance of combining the Fuzzy normalization approach with an entropy-based weighting technique to effectively estimate the factors’ relevant importance and weights and to refine the spatial scale of vulnerability index.
2. Sensitivity analysis of the vulnerability groups’ weights can potentially reveal important information about the spatial dominance of vulnerability groups more effectively. Sensitivity analysis of CVIs shows that Charleston County is more sensitive to socio-economic factors, whereas the physical factors contribute to a higher degree of vulnerability in Horry County.
3. PPCA method is more robust as determining the weights of the spatial factors is not required. The PPCA as a data imputation technique is a potential solution to assess the vulnerability at a precise resolution because the probability model of PPCA can interpolate missing values.
4. Validation of the results using historical events shows that both CVI-90 and PPCA preserve the accuracy of vulnerability estimation based on simultaneous information from biophysical and socio-economic factors, while the CVI-50 method underestimates the biophysical vulnerability of coastal hazards.

In conclusion, while the performance of CVI-90 outperformed other models to identify the vulnerable hotspots and socio-economic vulnerability, the results of our study show each of suggested methods still cannot solely provide comprehensive and precise information about the future of various vulnerability aspects. If any of the factors lack the accurate projections, the results from vulnerability assessment will be highly uncertain and may mislead decision makers when determining vulnerable places and suitable adaptation policies. This calls for the development of new holistic vulnerability assessment approaches in the future which focus on managing the deep uncertainty associated with socio-economic and hydroclimatic conditions. For example, advanced data driven algorithms for ranking vulnerability indictors and multi-variate copula based joint probability for developing stochastic CVI in future can provide room for keeping simultaneous information and more precise and case relevant factors for estimating vulnerability indices.

## Data Availability

The datasets used and/or analyzed during the current study available from the corresponding author on reasonable request.

## Abbreviations

CVI: Coastal vulnerability index
CVI-50: CVI with 50th percentile weight
CVI-90: CVI with 90th percentile weight
DEM: Digital elevation model
gSSURGO: Gridded soil survey geographic database
HVI: Hydroclimate vulnerability index
GEE: Google earth engine
GIS: Geographic information system
GPM: Global precipitation measurement mission
PC: Principal component
PCA: Principal component analysis
PPCA: Probabilistic principal component analysis
PVI: Physical vulnerability index
KMO: Kaiser–Meyer–Olkin
LRR: Linear rate of regression
MCDM: Multi criteria decision making
ML: Machin learning
NOAA: National Oceanic and Atmospheric Administration
NHC: National Hurricane Center
NHAS: No. of historical and archeological site
NWS: National Weather Service
NWR: National Wildlife Refugee
TOPSIS: Technique for order performance to ideal solution
SAR: Synthetic aperture radar
SC GAP: SC gap analysis program
SCDHEC: South Carolina Department of Health and Environmental
SoVI: Social vulnerability index
SEVI: Socio-economic vulnerability index
SLR: Sea level rise
SWH: Significant wave height
SLOSH: Sea, lake, and overland surges from hurricanes
USGS: US Geological Survey
USDA: United States Department of Agriculture
VH: Vertical–horizontal
VV: Vertical–vertical
